# Combined effect of diabetes and obesity on cancer risk in chronic kidney disease: a nationwide population-based study

**DOI:** 10.3389/fendo.2026.1708006

**Published:** 2026-02-23

**Authors:** Chang Seong Kim, Sang Heon Suh, Hong Sang Choi, Eun Hui Bae, Seong Kwon Ma, Jin Hyung Jung, Bongseong Kim, Kyung-Do Han, Soo Wan Kim

**Affiliations:** 1 Department of Internal Medicine, Chonnam National University Medical School, Gwangju, Republic of Korea; 2 Department of Internal Medicine, Chonnam National University Hospital, Gwangju, Republic of Korea; 3 Department of Biostatistics, College of Medicine, Catholic University of Korea, Seoul, Republic of Korea; 4 Department of Statistics and Actuarial Science, Soongsil University, Seoul, Republic of Korea

**Keywords:** body mass index, cancer risk, chronic kidney disease, diabetes mellitus, obesity, waist circumference

## Abstract

**Background:**

The effects of diabetes and obesity on cancer risk in patients with chronic kidney disease (CKD) remain unclear. We examined the independent and combined effects of diabetes, body mass index (BMI), and waist circumference (WC) on overall and site-specific cancer risk in CKD.

**Methods:**

Overall, data from 1,955,504 adults aged ≥ 20 years who underwent health checkups (2012–2017) were analyzed using the Korean National Health Insurance Service database. CKD was defined as an estimated glomerular filtration rate (eGFR) < 60 mL/min/1.73m² or proteinuria. Participants were stratified using glycemic status (normal, impaired fasting glucose [IFG], and diabetes), BMI (<18.5, 18.5–23, 23–25, 25–30, ≥30 kg/m²), and sex-specific WC categories.

**Results:**

During a median 7.7-year follow-up, 162,463 new cancer cases (8.3%) were identified among 1,955,504 participants with CKD. Compared with individuals with normal fasting glucose (NFG), those with IFG and diabetes had significantly higher overall cancer risk (adjusted HR [aHR] 1.025, 95% CI 1.012–1.037 and aHR 1.176, 95% CI 1.162–1.190, respectively). Higher BMI and WC were associated with cancer risk, with the highest categories showing aHRs of 1.080 (95% CI 1.056–1.105) and 1.128 (95% CI 1.106–1.152), respectively. Site-specific analyses showed diabetes was strongly associated with liver, gallbladder, and pancreatic cancers. Higher BMI and WC increased hepatobiliary, kidney, and female-specific cancer risks. Combined diabetes and obesity amplified risks, especially for gastrointestinal and female-specific cancers, while prostate cancer risk declined with lower BMI or WC regardless of diabetes.

**Conclusions:**

In this large CKD cohort, diabetes and obesity independently and jointly increased overall and site-specific cancer risk, particularly gastrointestinal and female-specific cancers.

## 1 Introduction

Chronic kidney disease (CKD) is a significant global public health burden, affecting > 690 million individuals worldwide (1, 2). Among the non-traditional complications of CKD, cancer occurs at a substantially higher frequency than in the general population (3, 4). Notably, the overall cancer incidence in CKD cohorts has been reported to be nearly fourfold higher than that of the general U.S. population (17.3 vs. 4.4 per 1,000 person-years) (5). With the growing prevalence of CKD worldwide, understanding modifiable risk factors for cancer in this population is of increasing importance. The global incidence and prevalence of diabetes and obesity have been increasing steadily. In 2019, approximately 9.3% of adults worldwide had diabetes, a figure projected to rise to 10.2% by 2030 (6). Concurrently, the rates of overweight and obesity have surged, posing a significant threat to global health. In 2021, an estimated 2.11 billion adults—almost half of the global adult population—were classified as overweight or obese (7). Among individuals with CKD, more than two-thirds are classified as overweight or obese (8, 9). Diabetes, obesity and CKD are centra components of the complex cardio-kidney-metabolic syndrome and share multiple interrelated risk factors (10). Given the substantial overlap in prevalence and the adverse clinical consequences observed in obese CKD patients with coexisting diabetes, evaluating the impact of diabetes and obesity on cancer development in patients with CKD is of considerable significance.

Diabetes and obesity are well-established risk factors for cardiovascular disease, but are increasingly related to cancer development. A recent multinational study reveals that distinct body shape phenotypes were positively associated with risks of 17 different cancer types (11). Moreover, epidemiological evidences indicate that these metabolic disorders may promote carcinogenesis through shared or distinct biological mechanisms (12, 13). The association between type 2 diabetes and cancer is believed to be mediated, at least in part, by metabolic dysfunction associated with obesity—particularly insulin resistance, hyperinsulinemia, insulin-like growth factor I, adipokines, cytokines, and alterations in the gut microbiome (14). However, larger-scale, population-based studies examining the independent or combined effects of diabetes and obesity on cancer risk within the CKD population remain limited.

Therefore, this study aims to investigate the association between diabetes and cancer risk, alongside the combined effects of diabetes with obesity—measured based on body mass index (BMI) or waist circumference (WC)—among patients with CKD, using a large nationwide population-based dataset from the Korean National Health Insurance Service (NHIS). Furthermore, risks for 24 site-specific cancers were evaluated to elucidate the differential influence of diabetes and obesity across various cancer types in individuals with CKD.

## 2 Patients and methods

### 2.1 Data source and study population

The data used in this study were obtained from the Korean National Health Insurance Service (NHIS) claims database, which compiles information from the NHIS and Medical Aid programs. The NHIS is a mandatory social insurance system that covers approximately 97% of the Korean population, while the remaining 3% are covered by the Medical Aid program (15). Consequently, data extracted from the NHIS database are considered representative of the entire South Korean population (approximately 50 million residents). All insured Koreans individuals aged > 40 years undergo biannual health checkups supported by the NHIS, while employed Koreans individuals aged > 20 years are required to undergo annual health checkups. During these examinations, body weight (kg), height (cm), WC (cm), and fasting serum glucose are measured. For this study, a subset of NHIS health checkup data collected between 2012 and 2022 was utilized.

The study protocol was approved by the Institutional Review Board (IRB) of Chonnam National University Hospital (CNUH-EXP-2025-263). All patient identification numbers were anonymized to ensure the protection of individual privacy. As only anonymized and de-identified data were used for analysis, the requirement for informed consent was waived by the IRB.

**Figure 1:**
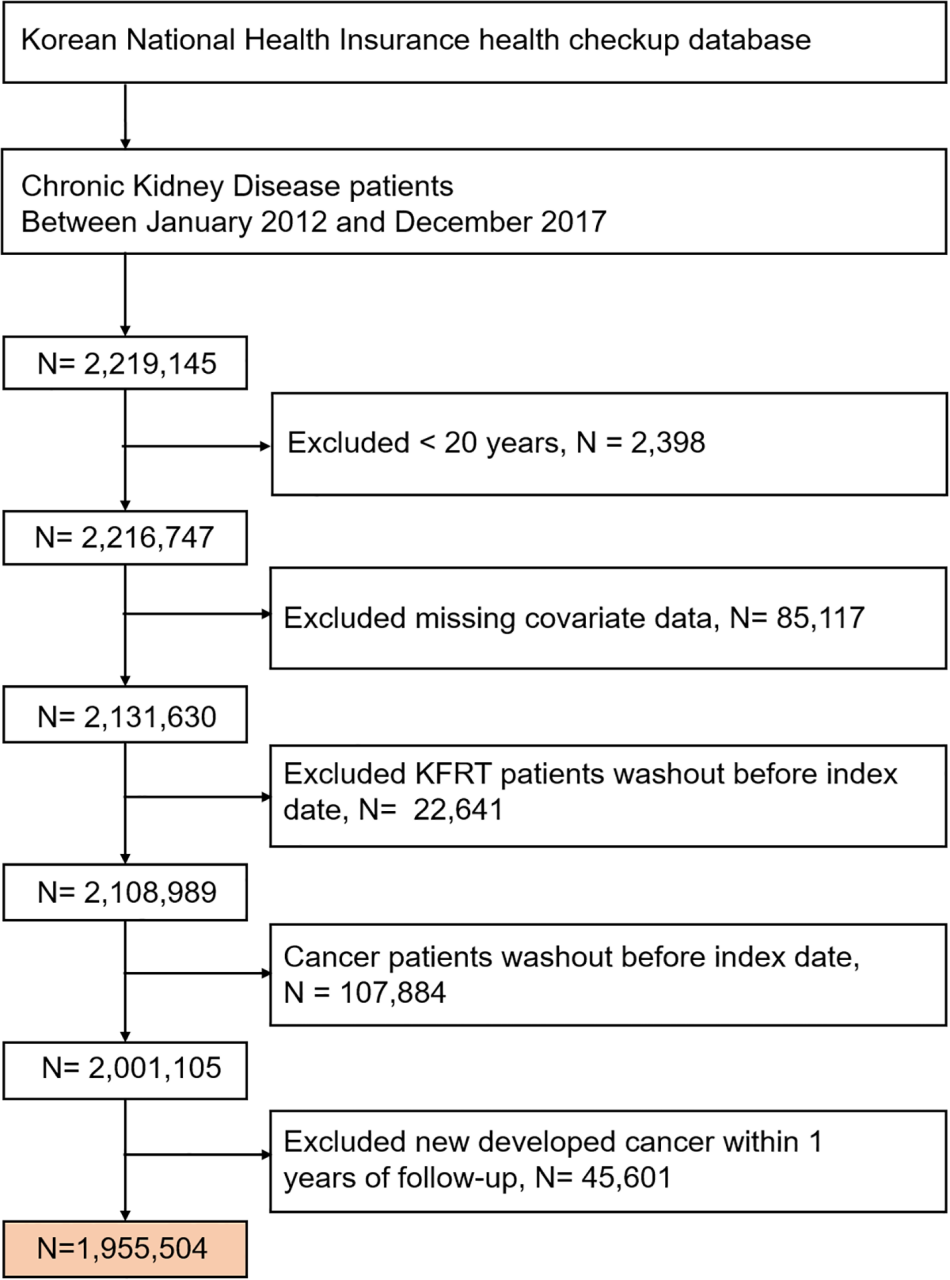
Flowchart of participant enrollment. KFRT, kidney failure with replacement therapy.

This study included data from 2,216,747 patients with CKD aged over 20 years who underwent at least one health examination between 2012 and 2017. CKD was defined as an estimated glomerular filtration rate (eGFR) of < 60 mL/min/1.73 m^2^, calculated using the CKD-Epidemiology Collaboration creatinine equation (16) or positive proteinuria. Individuals with a history of kidney failure requiring replacement therapy, a prior diagnosis of cancer, or missing covariate data were excluded. Additionally, individuals diagnosed with newly developed cancer within 1 year of follow-up were excluded to reduce potential bias from other predisposing or precipitating factors influencing the outcomes. Overall, 1,955,504 participants were included in the final analysis (**Figure 1**).

### 2.2 Definitions

BMI was calculated by dividing weight (kg) by the square of height (m^2^). Obesity was defined as a BMI ≥ 25 kg/m^2^. The participants were classified into five groups according to the World Health Organization recommendations for Asian populations: underweight (BMI < 18.5 kg/m^2^), normal (≥18.5 to < 23 kg/m^2^), overweight (≥ 23 to < 25 kg/m^2^), stage 1 obesity (≥ 25 to < 30 kg/m^2^), and stage 2 obesity (≥ 30 kg/m^2^) (17). The WC of each participant was measured at the midpoint between the rib cage and the iliac crest by a trained examiner. The patients were assigned into six groups based on 5-cm increments in WC as follows: < 80 cm for men and < 75 cm for women; 80–85 cm for men and 75–80 cm for women; 85–90 cm for men and 80–85 cm for women (reference group); 90–95 cm for men and 85–90 cm in women; 95–100 cm for men and 90–95 cm in women; and ≥ 100 cm for men and ≥ 95 cm for women. Additionally, all participants were classified into three groups based on glycemic status: normal fasting glucose (NFG), impaired fasting glucose (IFG), and diabetes. IFG was defined as a fasting plasma glucose level of 100–125 mg/dl. Diabetes was identified based on any of the following criteria: a documented clinical diagnosis (International Classification of Diseases Tenth Revision, Clinical Modification [ICD-10-CM] codes E11–14), recorded medical history of diabetes, or fasting serum glucose level ≥ 126 mg/dl as documented in the health examination database. Patients with type 1 diabetes, identified by claims under the ICD-10 code E10, were excluded from the study.

Hypertension was determined based on any of the following criteria: a documented diagnosis of hypertension (ICD-10-CM codes I10–13, I15), recorded history of antihypertensive medication use, or measured systolic blood pressure ≥ 140 mmHg or diastolic blood pressure ≥ 90 mmHg as recorded in the health examination database. Dyslipidemia was defined as the presence of ICD-10-CM code E78, a history of lipid-lowering medication use, or a total serum cholesterol level ≥ 240 mg/dl recorded in the health examination data.

Participants were additionally classified into three groups based on smoking status (non-smokers, former smokers, and current smokers). Alcohol consumption was also categorized into three groups: (non-drinkers, moderate drinkers, and heavy drinkers [≥ 30 g alcohol per day]). Regular exercise was defined as engaging in vigorous physical activity for at least 20 min per day for > 3 days, or moderate physical activity for at least 30 min per day for > 5 days during the previous week.

Proteinuria was assessed using a dipstick test. Fasting blood glucose, total cholesterol, triglycerides, high-density lipoprotein cholesterol, and low-density lipoprotein cholesterol levels (all in mg/dl) were measured in the fasting state. The quality of all laboratory tests was verified by the Korean Association for Laboratory Medicine, and the NHIS certified that participating hospitals were enrolled in the NHIS health checkup programs.

### 2.3 Study outcomes and follow-up

The primary outcome of this study was cancer incidence, identified using ICD-10 diagnosis codes recorded in the Korean NHIS database. The cancer types and corresponding codes included: oral cavity (C00 and C10–14), esophagus (C15), stomach (C16), colorectal (C18–20), liver (C22), gallbladder and bile duct (C23 and C24), pancreatic (C25), laryngeal (C32), lung (C33–34), skin (C43 and C44), breast (C50), cervical (C53), uterine (C54), ovarian (C56), prostate (C61), testicular (C62), kidney (C64), bladder (C67), brain and central nerve system (C71–72), thyroid (C73), Hodgkin’s lymphoma (C81), non-Hodgkin’s lymphoma (C82–86 and C96), multiple myeloma (C90), and leukemia (C91–C95). All participants were followed from the index date until the occurrence of cancer, death, censoring, or December 31, 2022. The total number of person-years of follow-up was then calculated. During a median follow-up period of 7.7 years, 162,463 participants developed cancer.

### 2.4 Statistical analyses

Continuous variables are presented as mean ± standard deviation, while categorical variables are presented as frequencies with corresponding proportions. Non-normally distributed variables are expressed as geometric means with 95% confidence intervals (CIs). Intergroup differences were assessed using the chi-squared test or analysis of variance, as appropriate. Cancer incidence rates are reported per 1,000 person-years. To evaluate cancer risk according to diabetes status and categories of BMI and WC, hazard ratios (HRs) with 95% CIs were calculated using multivariable Cox proportional hazard regression models. Model 1 was un-adjusted, while Model 2 was adjusted for age and sex. Model 3 was further adjusted for smoking status, alcohol consumption, regular exercise, low income, and previous history of hypertension and dyslipidemia including age and sex. Finally, the adjusted Cox model was used to examine the associations between diabetes status, cancer, and specific cancer types, stratified based on the five BMI groups and six WC groups, respectively. All statistical analyses were performed using SAS software (version 9.4; SAS Institute, Cary, NC, United States). Two-sided *P*-values < 0.05 were considered statistically significant.

## 3 Results

### 3.1 Baseline characteristics

**Table 1**; **Supplementary Tables S1**, **S2** present the baseline characteristics of 1,955,504 participants, stratified based on diabetes status, BMI (five levels), and WC (six levels). The mean baseline age was 53.9 years, and 47% of participants were male. The mean BMI and WC were 24.5 ± 3.5 kg/m^2^ and 83.1 ± 9.7 cm, respectively. Among the study population, 26.3% had IFG and 25.8% had diabetes. Among individuals with CKD, those with diabetes were more likely to be male, current smokers, and heavy alcohol consumers, and had a higher prevalence of hypertension and dyslipidemia compared to those with NFG. As BMI categories increased, the prevalence of hypertension, dyslipidemia, heavy alcohol consumption, and WC also increased proportionally. However, eGFR and proteinuria exhibited a U-shape association. Similarly, increasing WC was associated with higher proportions of heavy alcohol drinkers, hypertension, dyslipidemia, and elevated BMI, while eGFR and proteinuria again demonstrated a U-shape pattern.

**Table 1:** Baseline characteristics of the study population by diabetes mellitus status.

Characteristics	Total	Diabetes Mellitus status			
		NFG	IFG	Diabetes Mellitus	*P* value
Number	1955504	936868	513562	505074	
Age, years, mean ± SD	53.9 ± 21.13	56.38 ± 16.02	60.67 ± 13.86	64.08 ± 11.81	< 0.001
Age group, years (%)					< 0.001
20–40	198838(10.17)	149848(15.99)	36011(7.01)	12979(2.57)	
40–65	953860(48.78)	462087(49.32)	260642(50.75)	231131(45.76)	
≥ 65	802806(41.05)	324933(34.68)	216909(42.24)	260964(51.67)	
Male (%)	918693(46.98)	381404(40.71)	260024(50.63)	277265(54.9)	< 0.001
Low income (%)	426491(21.81)	200692(21.42)	109068(21.24)	116731(23.11)	< 0.001
Smoking (%)					< 0.001
Non	1291256(66.03)	658991(70.34)	327335(63.74)	304930(60.37)	
Former	323304(16.53)	129604(13.83)	94349(18.37)	99351(19.67)	
Current	340944(17.44)	148273(15.83)	91878(17.89)	100793(19.96)	
Alcohol consumption					< 0.001
None (%)	1262586(64.57)	610140(65.13)	315190(61.37)	337256(66.77)	
Moderate (%)	573318(29.32)	283837(30.3)	160015(31.16)	129466(25.63)	
Heavy (%)	119600(6.12)	42891(4.58)	38357(7.47)	38352(7.59)	
Regular exercise (%)	388059(19.84)	185902(19.84)	102923(20.04)	99234(19.65)	< 0.001
Hypertension (%)	1104581(56.49)	408743(43.63)	300721(58.56)	395117(78.23)	< 0.001
Dyslipidemia (%)	772091(39.48)	277570(29.63)	201183(39.17)	293338(58.08)	< 0.001
eGFR <60 ml/min/1.73m^2^ (%)	1192704(60.99)	542931(57.95)	327320(63.74)	322453(63.84)	< 0.001
Urine proteinuria (%)	860222(43.99)	424881(45.35)	207250(40.36)	228091(45.16)	< 0.001
Height, cm	160.86 ± 9.58	160.73 ± 9.5	161.16 ± 9.75	160.79 ± 9.56	< 0.001
Weight, kg	63.68 ± 12.35	61.79 ± 11.88	65.04 ± 12.57	65.79 ± 12.46	
BMI, kg/m^2^, mean ± SD	24.5 ± 3.53	23.81 ± 3.41	24.91 ± 3.48	25.34 ± 3.57	< 0.001
WC, cm, mean ± SD	83.1 ± 9.71	80.53 ± 9.58	84.17 ± 9.21	86.77 ± 9.03	< 0.001
Fasting glucose, mg/dL, mean ± SD	109.24 ± 36.92	88.91 ± 7.4	108.57 ± 6.79	147.62 ± 53.74	< 0.001
SBP, mmHg, mean ± SD	126.9 ± 16.49	123.85 ± 16.05	128.65 ± 16.12	130.8 ± 16.59	< 0.001
DBP, mmHg, mean ± SD	77.68 ± 10.68	76.51 ± 10.5	79.06 ± 10.69	78.44 ± 10.77	< 0.001
Total cholesterol, mg/dL, mean ± SD	196.4 ± 41.88	196.91 ± 39.11	203.79 ± 41.55	187.93 ± 45.5	< 0.001
High-density lipoprotein, mg/dL, mean ± SD	53.33 ± 16.02	55.37 ± 16.47	53.51 ± 15.75	49.39 ± 14.69	< 0.001
Low-density lipoprotein, mg/dL, mean ± SD	114.49 ± 38.07	116.46 ± 35.9	120.19 ± 38.28	105.03 ± 40.03	< 0.001
Triglyceride, mg/dL, (25^th^ 75^th^)	124.01(123.92-124.11)	108.6(108.48-108.72)	132.38(132.19-132.58)	148.44(148.21-148.66)	< 0.001

eGFR, estimated glomerular filtration rate; BMI, body mass index; WC, waist circumference; SBP, systolic blood pressure; DBP, diastolic blood pressure; SD, standard deviation.

### 3.2 Associations of diabetes and obesity with overall and site-specific cancer risks

During a median follow-up period of 7.7 years, 162,463 individuals (8.3%) were diagnosed with cancer. **Table 2** presents the associations between diabetes status, obesity-related metrics, and overall cancer risk. Cancer incidence rates increased progressively across glycemic categories, with 9.3, 11.5, and 14.9 cases per 1,000 person-years observed in individuals with NFG, IFG, and diabetes, respectively. After adjusting for potential confounders, individuals with IFG and diabetes demonstrated a significantly increased risk of overall cancer compared to those with NFG, with adjusted HRs of 1.025 (95% CI, 1.012–1.037) and 1.176 (95% CI, 1.162–1.190), respectively. When participants were stratified based on BMI and WC categories, those with a BMI of ≥ 30 kg/m^2^ demonstrated a significantly increased risk of overall cancer compared to those in the reference group (BMI 18.5–23.0 kg/m^2^), with an adjusted HR of 1.080 (95% CI, 1.056–1.105). In the WC categories, overall cancer risk increased progressively with higher WC levels compared to those of the reference group. The peak risk was observed in the highest WC category (≥ 100/≥95 cm), with an adjusted HR of 1.128 (95% CI, 1.106–1.152).

**Table 2:** Incidence rates and hazard ratios of cancer by diabetes status, body mass index and waist circumference.

Group	Number	Cancer	Follow-up duration, person-year	Incidence rate, per 1000 person-year	Model 1, HR (95% CI) ^a^	Model 2, HR (95% CI) ^b^	Model 3, HR (95% CI) ^c^
DM status							
NFG	936868	65955	7026492	9.39	reference	reference	reference
IFG	513562	43425	3772333	11.51	1.228(1.214,1.243)	1.03(1.018,1.043)	1.025(1.012,1.037)
DM	505074	53083	3554676	14.93	1.596(1.578,1.614)	1.19(1.176,1.204)	1.176(1.162,1.19)
BMI group, kg/m^2^							
	62179	3812	423720	9.00	0.842(0.815,0.87)	1.017(0.984,1.052)	1.011(0.978,1.045)
18.5–23	596875	46385	4330257	10.71	reference	reference	reference
23–25	469846	41101	3466882	11.86	1.106(1.091,1.121)	0.986(0.973,0.999)	0.985(0.972,0.998)
25–30	700046	61587	5194623	11.86	1.106(1.093,1.119)	1.008(0.996,1.02)	1.003(0.99,1.015)
≥ 30	126558	9578	938018	10.21	0.953(0.933,0.975)	1.101(1.076,1.125)	1.08(1.056,1.105)
WC group, cm, (male/female)							
<80/<75	476365	30545	3514558	8.69	0.726(0.715,0.737)	0.99(0.975,1.005)	0.997(0.982,1.013)
80–85/75–80	401285	32657	2961065	11.03	0.921(0.907,0.934)	0.98(0.966,0.995)	0.983(0.969,0.998)
85–90/80–85	436958	38519	3215870	11.98	reference	reference	reference
90–95/85–90	324680	30873	2373540	13.01	1.086(1.07,1.103)	1.049(1.033,1.065)	1.045(1.029,1.061)
95–100/90–95	181355	17398	1317831	13.20	1.103(1.083,1.123)	1.08(1.061,1.1)	1.072(1.053,1.092)
≥ 100/≥ 95	134861	12471	970635	12.85	1.075(1.053,1.096)	1.143(1.12,1.167)	1.128(1.106,1.152)

^a^Model 1, Non-adjusted. ^b^Model 2, adjusted for age and sex. ^c^Model 3, Adjusted for age, sex, smoking, alcohol drinking, regular exercise, low income, previous history of hypertension and dyslipidemia. DM, diabetes mellitus; BMI, body mass index; WC, waist circumference; HR, hazard ratio; CI, confidential interval; NGF, normal fasting glucose; IFG, impaired fasting glucose.

To further elucidate the associations between diabetes status, obesity-related parameters, and site-specific cancer risk, comprehensive analyses were conducted across 24 cancer types, including solid cancers, hematologic malignancies, and sex-specific cancers in men and women (**Supplementary Tables S3**-**S5**). Among patients with CKD, diabetes was significantly associated with an increased risk of liver, gallbladder and biliary tract, and pancreatic cancers (adjusted HRs: 1.980, 1.369, and 1.433, respectively). Higher BMI was associated with increased risks of hepatobiliary, kidney, and female-specific cancers, with the strongest association observed for uterine cancer (adjusted HR, 3.284; 95% CI, 2.802–3.849) in individuals with BMI ≥ 30 kg/m². Similarly, elevated WC (≥ 100/≥ 95 cm) was associated with higher risks of liver, kidney, and uterine cancers (adjusted HRs: 1.506, 1.374, and 1.875, respectively).

### 3.3 Combined effects of the diabetes status and BMI or WC level on the risk of cancer

**Figure 2:**
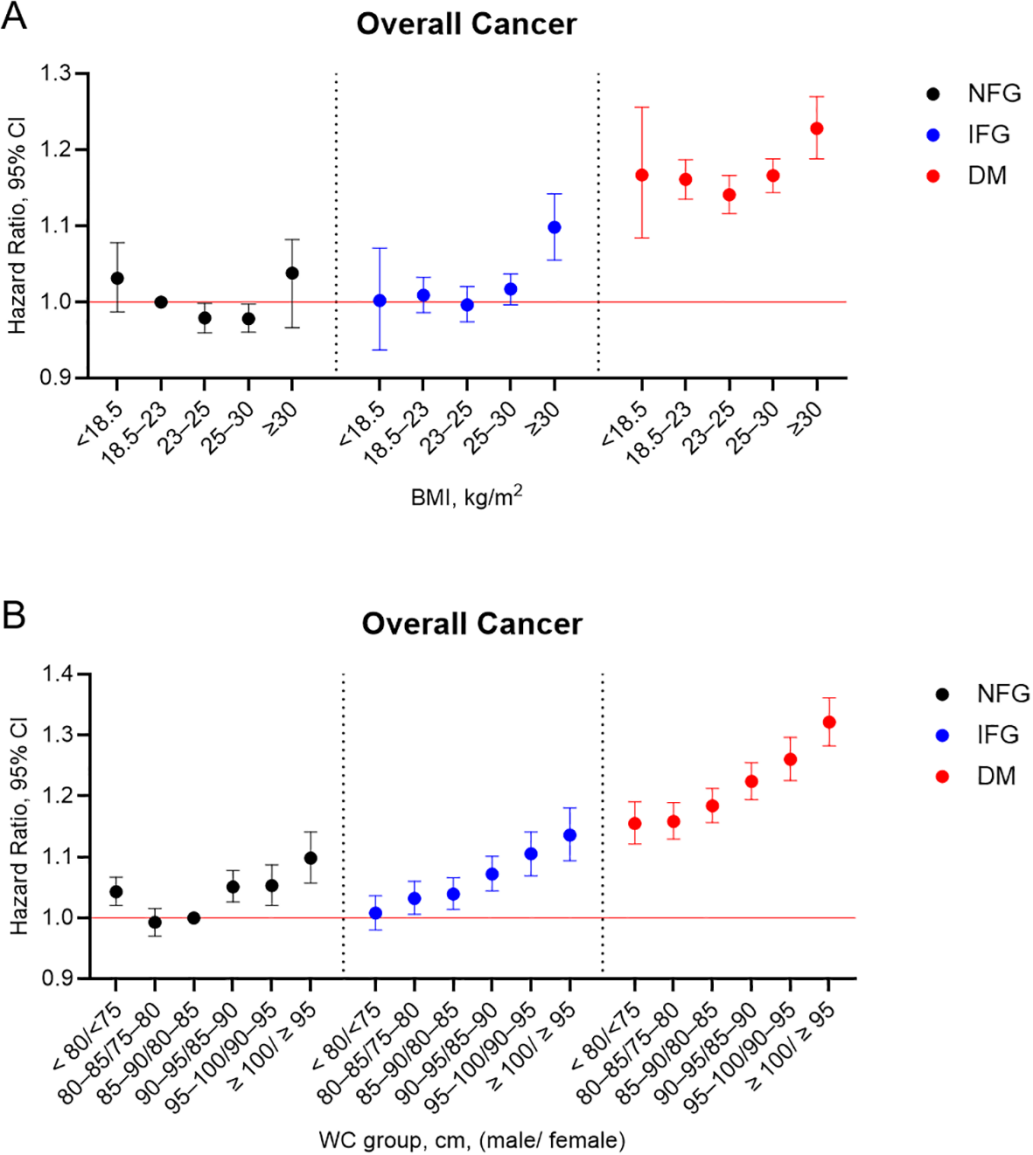
Adjusted hazard ratios for overall cancer incidence based on diabetes status and **(A)** body mass index or **(B)** waist circumference. Vertical lines indicate 95% confidence intervals. Models were adjusted for age, sex, smoking, alcohol consumption, regular exercise, low income, and previous history of hypertension and dyslipidemia. DM, diabetes mellitus; NFG, normal fasting glucose; IFG, impaired fasting glucose.

We analyzed the incidence rates and adjusted HRs for overall cancer based on BMI or WC categories, stratified according to diabetes status (**Figure 2A**). With participants who had NFG and a BMI of 18.5–23 kg/m² serving as the reference group, those in the NFG group with a BMI of 23–30 kg/m² exhibited a significantly lower risk of overall cancer. Among individuals with IFG, a significantly elevated cancer risk was observed only in those with a BMI ≥ 30 kg/m² (adjusted HR, 1.098; 95% CI, 1.055–1.142). In contrast, participants with diabetes exhibited significantly increased cancer risks across all BMI categories, with the highest risk observed in those with a BMI ≥ 30 kg/m² (adjusted HR, 1.228; 95% CI, 1.188–1.270). With participants who had NFG and reference WC serving as the baseline, a U-shaped association between WC and overall cancer risk was observed within the NFG group (**Figure 2B**). In contrast, among individuals with IFG and diabetes, cancer risk increased linearly with rising WC levels. In the diabetes group, participants with the lowest WC (< 80/< 75 cm) had a higher cancer risk than those in the IFG group with the highest WC (≥ 100/≥ 95 cm). Additionally, we assessed the combined effects of diabetes status and obesity-related parameters on the risk of 24 site-specific cancers (Data not shown). Among individuals with diabetes, a trend of increasing cancer risk was observed with rising levels of BMI and WC, particularly for cancers of the stomach, colorectum, liver, gallbladder and bile duct, pancreas, breast, cervix, uterus, and ovary. In contrast, the risk of prostate cancer decreased in individuals with lower BMI or WC, regardless of diabetes status.

## 4 Discussion

In this large population-based cohort study of Korean adults with CKD, both diabetes and elevated levels of BMI and WC were independently and jointly associated with an increased risk of overall and site-specific cancers. Our analysis uniquely incorporates diabetes status together with both BMI and WC, enabling a detailed assessment of metabolic risk patterns across 24 site-specific cancers in patients with CKD. The combination of diabetes with higher BMI or WC was associated with substantially elevated risks for several gastrointestinal and female-specific cancers, including cancers of the stomach, colorectum, liver, biliary tract, pancreas, breast, cervix, uterus, and ovary. In contrast, the risk of prostate cancer was lower among participants with lower BMI or WC, irrespective of diabetes status.

These findings build upon prior studies demonstrating that individuals with diabetes and obesity are more susceptibility to an increased risk of overall cancer, alongside several specific cancer types, such as colorectal, liver, pancreatic, and endometrial cancers, in the general population (11, 18–22). In this study, similar associations have also been observed in patients with CKD. A previous meta-analysis of 151 cohorts, including over 32 million individuals, shows that participants with type 2 DM had a 15% higher risk of overall cancer incidence compared with those without diabetes, although substantial variation was revealed across cancer types (20). Furthermore, epidemiologic studies investigating the relationship between obesity and cancer have reveal that elevated BMI is significantly associated with increased risks of cancers of the stomach, colorectum, liver, gallbladder, pancreas, and kidney, with a clear positive dose-response association (23). In regions with a high prevalence of obesity, between 4% and 9% of the cancer burden has been attributed to a BMI of ≥ 25kg/m^2^, consistent with the associations revealed in our CKD population (24). A meta-analysis of European prospective cohort studies similarly reveals that BMI, WC, hip circumference, and waist-to-hip ratio were comparably associated with an increased risk of obesity-related cancers, including colorectal cancer in older adults (25). In addition, a large prospective cohort study involving 3.5 million adults in Spain reveals that BMI and WC demonstrate a similar relationship with cancer risk (26). Consistent with these findings, our study revealed an approximately 8%–13% higher incidence of cancer among patients with obesity and CKD. When BMI exceeded 25 kg/m^2^ and WC reached ≥ 100 in men or ≥ 95 cm in women, the HR increased for obesity-related cancers, including those of the colorectum, liver and kidney. WC shows a stronger association than BMI with breast, endometrial, cervical, and ovarian cancers. WC is commonly used as a surrogate marker for abdominal fat mass and has been revealed to more accurately predict intra-abdominal or visceral adiposity compared to BMI (27). This may reflect a closer association between body shape phenotypes and the risk of site-specific cancers, particularly female-specific obesity-related cancers in patients with CKD (11).

Several biological mechanisms may underlie the association between diabetes, obesity, and an increased cancer risk. Metabolic abnormalities commonly observed in individuals with type 2 diabetes and obesity—including hyperinsulinemia and hyperlipidemia—are implicated in promoting cancer development and progression (12, 28). Hyperinsulinemia enhances insulin-like growth factor signaling, which subsequently activates the phosphatidylinositol 3−kinase/Akt/mammalian target of rapamycin (mTOR) and mitogen-activated protein kinase pathways, thereby promoting cancer cell proliferation, survival, migration, and drug resistance (29, 30). Furthermore, elevated cholesterol and non-esterified fatty acids under hyperlipidemic conditions may activate Akt/mTOR and Akt/GSK3β/β-catenin pathways, alongside enhancing adenosine triphosphate production through β oxidation, thereby promoting cancer cell proliferation and tumor progression (13, 28). In addition, in the context of obesity, adipose tissue dysfunction, altered endogenous hormone levels—including hyperinsulinemia—and adipokine-mediated chronic inflammation are recognized mechanisms that facilitate carcinogenesis (31). These metabolic alterations may contribute to cancer progression through direct and indirect pathways.

To evaluate the combined effect of diabetes and obesity on cancer risk, we examine their association with cancer incidence in patients with CKD, considering their shared contribution through metabolic alterations. Our findings reveal a synergistic increase in obesity-related cancer risk among individuals with diabetes compared to those with prediabetes. BMI exhibits a U-shaped association with cancer risk as diabetes progresses, whereas WC reveals a consistent linear relationship with cancer risk across the prediabetic and diabetic groups. Limited evidence exists on how obesity influences cancer risk based on diabetes status in patients with CKD; however, previous research reveals that greater WC is associated with an increased risk of colorectal cancer. This association is stronger among individuals with more advanced diabetes, which is consistent with our findings (32). Additionally, a meta-analysis of the European Prospective Investigation into Cancer and Nutrition and the UK Biobank cohorts shows that the combined effect of high BMI and type 2 diabetes on cancer risk exceeded the sum of their individual effects (33). These findings suggest that preventing obesity may be especially important for reducing cancer risk in individuals with diabetic kidney disease, compared to those with CKD without diabetes. Further large-scale prospective studies are needed to evaluate whether weight loss and glycemic control can effectively reduce cancer risk in patients with CKD.

A major strength of this study is the large, nationwide cohort, comprising 14 million person-years of follow-up. This extensive dataset enabled the assessment of 24 site-specific cancer risks, allowing for a detailed analysis of the independent and combined effects of diabetes and obesity in patients with CKD. However, the study has some limitations. First, BMI and WC were measured only once, which may not reflect changes in obesity status over time. Such non-differential exposure misclassification during the follow-up period may have attenuated the observed associations, potentially underestimating the true cancer risks. Future studies with repeated or time-updated measurements are needed to more accurately evaluate how longitudinal changes in diabetes status and obesity influence cancer development in patients with CKD. Second, the median follow-up period of 7.7 years may be insufficient to fully assess cancer risk, given the long latency period typically associated with cancer development. Therefore, extended follow-up is necessary to confirm the association between diabetes, obesity, and cancer risk. Third, in patients with advanced CKD, factors such as edema may increase body weight and result in an overestimation of BMI, a limitation not addressed in this study. Fourth, we were unable to adjust for medications such as antidiabetic or lipid-lowering agents, which may independently influence cancer risk. Given the medication use is intrinsically linked to the definition and severity of diabetes status itself, comprehensive adjustment was not feasible; therefore, residual confounding from medication use may remain (34, 35). Fifth, because both CKD and cancer are strongly age-related conditions, age may serve as a significant confounding factor. Although age was fully adjusted for in our multivariable models, residual confounding may persist, and this should be considered when interpreting the findings. Finally, owing to the observational design of the study, a direct causal relationship could not be established.

## 5 Conclusions

This large population-based cohort study of Korean adults with CKD shows that diabetes, elevated BMI, and increased WC were each independently and jointly associated with a higher risk of overall and site-specific cancers. Importantly, this study is the first to comprehensively evaluate the combined influence of diabetes and obesity on 24 different cancer types in a CKD population. The coexistence of diabetes with elevated BMI or WC was particularly associated with substantially increased risks of several gastrointestinal and female-specific cancers. These findings highlight the need for targeted cancer prevention strategies in high-risk CKD populations.

## Data Availability

The original contributions presented in the study are included in the article/**Supplementary Material**. Further inquiries can be directed to the corresponding authors.
