# Supplementary material for: Combined effect of diabetes and obesity on cancer risk in chronic kidney disease: a nationwide population-based study
Source: Front Endocrinol (Lausanne). 2026 Feb 23;17:1708006. doi: 10.3389/fendo.2026.1708006 (PMC12968020; doi:10.3389/fendo.2026.1708006)
Supplement: Supplementary file 1 [file Table1.docx]

**Table S1.** Baseline characteristics of the study population according to the body mass index levels

| Characteristics | Total | BMI, kg/m^2^ | | | | |  |
| --- | --- | --- | --- | --- | --- | --- | --- |
|  |  | <18.5 | 18.5–23 | 23–25 | 25–30 | ≥30 | *P* value |
| Number | 1955504 | 62179 | 596875 | 469846 | 700046 | 126558 |  |
| Age, years, mean ± SD | 53.9±21.13 | 58.5±16.3 | 61.1±13.5 | 60.5±13.26 | 55.3±14.94 | 53.9±21.13 | < 0.001 |
| Age group, years (%) |  |  |  |  |  |  | < 0.001 |
| 20–40 | 198838(10.17) | 19453(31.29) | 79898(13.39) | 30554(6.5) | 47969(6.85) | 20964(16.56) |  |
| 40–65 | 953860(48.78) | 19767(31.79) | 277800(46.54) | 232532(49.49) | 357774(51.11) | 65987(52.14) |  |
| ≥ 65 | 802806(41.05) | 22959(36.92) | 239177(40.07) | 206760(44.01) | 294303(42.04) | 39607(31.3) |  |
| Male (%) | 918693(46.98) | 18300(29.43) | 235467(39.45) | 234928(50) | 370125(52.87) | 59873(47.31) | < 0.001 |
| Low income (%) | 426491(21.81) | 14513(23.34) | 133954(22.44) | 100438(21.38) | 148894(21.27) | 28692(22.67) | < 0.001 |
| Smoking (%) |  |  |  |  |  |  | < 0.001 |
| Non | 1291256(66.03) | 45700(73.5) | 419805(70.33) | 305353(64.99) | 439362(62.76) | 81036(64.03) |  |
| Former | 323304(16.53) | 4912(7.9) | 75519(12.65) | 84809(18.05) | 138289(19.75) | 19775(15.63) |  |
| Current | 340944(17.44) | 11567(18.6) | 101551(17.01) | 79684(16.96) | 122395(17.48) | 25747(20.34) |  |
| Alcohol consumption |  |  |  |  |  |  | < 0.001 |
| None (%) | 1262586(64.57) | 41369(66.53) | 396772(66.47) | 304309(64.77) | 441046(63) | 79090(62.49) |  |
| Moderate (%) | 573318(29.32) | 18066(29.05) | 170788(28.61) | 138078(29.39) | 209417(29.91) | 36969(29.21) |  |
| Heavy (%) | 119600(6.12) | 2744(4.41) | 29315(4.91) | 27459(5.84) | 49583(7.08) | 10499(8.3) |  |
| Regular exercise (%) | 388059(19.84) | 7441(11.97) | 114559(19.19) | 101941(21.7) | 143042(20.43) | 21076(16.65) | < 0.001 |
| Hypertension (%) | 1104581(56.49) | 19209(30.89) | 262079(43.91) | 266614(56.74) | 462720(66.1) | 93959(74.24) | < 0.001 |
| Dyslipidemia (%) | 772091(39.48) | 10740(17.27) | 180859(30.3) | 190152(40.47) | 325823(46.54) | 64517(50.98) | < 0.001 |
| eGFR <60 ml/min/1.73m^2^ (%) | 1192704(60.99) | 28174(45.31) | 351857(58.95) | 305905(65.11) | 443187(63.31) | 63581(50.24) | < 0.001 |
| Urine proteinuria (%) | 860222(43.99) | 36668(58.97) | 272364(45.63) | 187234(39.85) | 293977(41.99) | 69979(55.29) | < 0.001 |
| Height, cm | 160.86±9.58 | 160±9.1 | 160.18±9.11 | 160.9±9.48 | 161.34±9.81 | 161.72±10.77 | < 0.001 |
| Weight, kg | 63.68±12.35 | 44.66±5.68 | 54.64±7.01 | 62.25±7.5 | 70.18±9.22 | 84.92±13.23 |  |
| BMI, kg/m^2^, mean ± SD | 24.5±3.53 | 17.38±0.95 | 21.23±1.2 | 23.95±0.57 | 26.86±1.33 | 32.32±2.49 | < 0.001 |
| WC, cm, mean ± SD | 83.1±9.71 | 66.9±6.5 | 75.63±6.68 | 82.38±5.88 | 88.55±6.41 | 98.77±8.1 | < 0.001 |
| Fasting glucose, mg/dL, mean ± SD | 109.24±36.92 | 99.74±36.5 | 104.48±35.97 | 108.79±35.68 | 112.56±36.79 | 119.62±42.05 | < 0.001 |
| SBP, mmHg, mean ± SD | 126.9±16.49 | 117.37±17.01 | 122.89±16.44 | 126.99±15.77 | 129.82±15.68 | 134.09±16.85 | < 0.001 |
| DBP, mmHg, mean ± SD | 77.68±10.68 | 72.95±10.57 | 75.3±10.32 | 77.45±10.18 | 79.38±10.44 | 82.71±11.78 | < 0.001 |
| Total cholesterol, mg/dL, mean ± SD | 196.4±41.88 | 185.09±38.24 | 193.25±40.39 | 197.09±41.84 | 198.66±42.79 | 201.71±43.6 | < 0.001 |
| High-density lipoprotein, mg/dL, mean ± SD | 53.33±16.02 | 62.7±18.86 | 56.95±17.04 | 52.59±15.46 | 50.6±14.59 | 49.58±13.99 | < 0.001 |
| Low-density lipoprotein, mg/dL, mean ± SD | 114.49±38.07 | 102.92±34.09 | 112.43±36.52 | 115.92±38.11 | 115.93±39.07 | 116.56±39.94 | < 0.001 |
| Triglyceride, mg/dL, (25^th^ 75^th^) | 124.01(123.92-124.11) | 84.56(84.23-84.9) | 103.01(102.87-103.16) | 125.13(124.93-125.32) | 142.58(142.4-142.75) | 160.6(160.13-161.07) | < 0.001 |

Abbreviations: eGFR, estimated glomerular filtration rate; BMI, body mass index; WC, waist circumference; SBP, systolic blood pressure; DBP, diastolic blood pressure; SD, standard deviation.

**Table S2.** Baseline characteristics of the study population according to the waist circumference levels

| Characteristics | Total | WC group, cm, (male/ female) | | | | | |  |  |
| --- | --- | --- | --- | --- | --- | --- | --- | --- | --- |
|  |  | < 80/<75 | 80–85/75–80 | 85–90/80–85 | 90–95/85–90 | 95–100/90–95 | ≥ 100/ ≥ 95 | | *P* value |
| Number | 1955504 | 476365 | 401285 | 436958 | 324680 | 181355 | 134861 | |  |
| Age, years, mean ± SD | 53.9±21.13 | 53.87±16.73 | 59.59±13.95 | 61.56±13.27 | 62.41±13.13 | 62.8±13.52 | 60.96±14.97 | | < 0.001 |
| Age group, years (%) |  |  |  |  |  |  |  | | < 0.001 |
| 20–40 | 19453(31.29) | 96248(20.2) | 32808(8.18) | 26202(6) | 18075(5.57) | 11381(6.28) | 14124(10.47) | |  |
| 40–65 | 19767(31.79) | 242969(51) | 211763(52.77) | 214224(49.03) | 150119(46.24) | 78074(43.05) | 56711(42.05) | |  |
| ≥ 65 | 22959(36.92) | 137148(28.79) | 156714(39.05) | 196532(44.98) | 156486(48.2) | 91900(50.67) | 64026(47.48) | |  |
| Male (%) | 18300(29.43) | 173305(36.38) | 205046(51.1) | 224247(51.32) | 170141(52.4) | 86539(47.72) | 59415(44.06) | | < 0.001 |
| Low income (%) | 14513(23.34) | 107756(22.62) | 86508(21.56) | 93038(21.29) | 69240(21.33) | 39272(21.65) | 30677(22.75) | | < 0.001 |
| Smoking (%) |  |  |  |  |  |  |  | | < 0.001 |
| Non | 45700(73.5) | 344488(72.32) | 256497(63.92) | 278444(63.72) | 203507(62.68) | 118555(65.37) | 89765(66.56) | |  |
| Former | 4912(7.9) | 52272(10.97) | 69957(17.43) | 82378(18.85) | 64362(19.82) | 32810(18.09) | 21525(15.96) | |  |
| Current | 11567(18.6) | 79605(16.71) | 74831(18.65) | 76136(17.42) | 56811(17.5) | 29990(16.54) | 23571(17.48) | |  |
| Alcohol consumption |  |  |  |  |  |  |  | | < 0.001 |
| None (%) | 41369(66.53) | 304664(63.96) | 253049(63.06) | 281725(64.47) | 209899(64.65) | 121555(67.03) | 91694(67.99) | |  |
| Moderate (%) | 18066(29.05) | 150034(31.5) | 123688(30.82) | 127183(29.11) | 91961(28.32) | 47107(25.98) | 33345(24.73) | |  |
| Heavy (%) | 2744(4.41) | 21667(4.55) | 24548(6.12) | 28050(6.42) | 22820(7.03) | 12693(7) | 9822(7.28) | |  |
| Regular exercise (%) | 7441(11.97) | 95088(19.96) | 87439(21.79) | 91065(20.84) | 62521(19.26) | 31467(17.35) | 20479(15.19) | | < 0.001 |
| Hypertension (%) | 19209(30.89) | 162711(34.16) | 208053(51.85) | 268487(61.44) | 223253(68.76) | 134964(74.42) | 107113(79.42) | | < 0.001 |
| Dyslipidemia (%) | 10740(17.27) | 116506(24.46) | 148549(37.02) | 188769(43.2) | 153591(47.31) | 92515(51.01) | 72161(53.51) | | < 0.001 |
| eGFR <60 ml/min/1.73m^2^ (%) | 28174(45.31) | 245331(51.5) | 248832(62.01) | 285107(65.25) | 213462(65.75) | 118613(65.4) | 81359(60.33) | | < 0.001 |
| Urine proteinuria (%) | 36668(58.97) | 249257(52.32) | 171024(42.62) | 174095(39.84) | 129508(39.89) | 73819(40.7) | 62519(46.36) | | < 0.001 |
| Height, cm | 160±9.1 | 159.87±8.71 | 160.92±9.31 | 161.02±9.68 | 161.51±9.98 | 161.21±10.28 | 161.65±10.74 | | < 0.001 |
| Weight, kg | 44.66±5.68 | 53.79±7.86 | 60.73±8.63 | 64.54±9.47 | 68.72±10.36 | 72.47±11.44 | 80.58±14.94 | |  |
| BMI, kg/m^2^, mean ± SD | 17.38±0.95 | 21±2.18 | 23.37±1.97 | 24.8±2.07 | 26.23±2.21 | 27.75±2.42 | 30.65±3.54 | | < 0.001 |
| WC, cm, mean ± SD | 66.9±6.5 | 71.01±4.95 | 79.59±2.9 | 84.48±2.9 | 89.4±2.83 | 94.05±2.87 | 101.84±5.5 | | < 0.001 |
| Fasting glucose, mg/dL, mean ± SD | 99.74±36.5 | 100.55±32.3 | 107.33±35.46 | 110.61±36.53 | 113.59±37.93 | 116.25±39.46 | 121.25±43.73 | | < 0.001 |
| SBP, mmHg, mean ± SD | 117.37±17.01 | 120.57±16.1 | 126.05±15.85 | 128.29±15.74 | 130.06±15.83 | 131.47±16.03 | 133.63±16.81 | | < 0.001 |
| DBP, mmHg, mean ± SD | 72.95±10.57 | 74.59±10.3 | 77.21±10.29 | 78.28±10.33 | 79.18±10.54 | 79.92±10.78 | 81.39±11.58 | | < 0.001 |
| Total cholesterol, mg/dL, mean ± SD | 185.09±38.24 | 192.74±39.34 | 197.11±41.51 | 197.88±42.43 | 197.58±42.98 | 197.55±43.57 | 197.96±44.11 | | < 0.001 |
| High-density lipoprotein, mg/dL, mean ± SD | 62.7±18.86 | 59.41±17.54 | 53.56±15.82 | 51.5±14.93 | 50.25±14.48 | 49.75±14.2 | 49.37±14.03 | | < 0.001 |
| Low-density lipoprotein, mg/dL, mean ± SD | 102.92±34.09 | 111.67±35.62 | 116.02±37.78 | 116.02±38.52 | 115.01±39.2 | 114.29±39.83 | 113.89±40.1 | | < 0.001 |
| Triglyceride, mg/dL, (25^th^ 75^th^) | 84.56(84.23-84.9) | 93.48(93.34-93.63) | 120.25(120.05-120.45) | 133.81(133.6-134.03) | 143.65(143.39-143.91) | 150.08(149.72-150.44) | 156.57(156.15-157) | | < 0.001 |

Abbreviations: eGFR, estimated glomerular filtration rate; BMI, body mass index; WC, waist circumference; SBP, systolic blood pressure; DBP, diastolic blood pressure; SD, standard deviation.

**Table S3.** Incidence rates and hazard ratios of site-specific cancers by diabetes status

| Cancer site | DM status | Number | Cancer | Follow-up Duration, Person-year | Incidence Rate, Per 1000 person-year | Model 1, HR (95% CI) ^a^ | Model 2, HR (95% CI) ^b^ | Model 3, HR (95% CI) ^c^ |
| --- | --- | --- | --- | --- | --- | --- | --- | --- |
| Lips, oral cavity and pharynx (C00-C14) | NFG | 936868 | 1017 | 7222155 | 0.14 | 1(Ref.) | 1(Ref.) | 1(Ref.) |
|  | IFG | 513562 | 768 | 3895590 | 0.20 | 1.403(1.278,1.541) | 1.141(1.039,1.254) | 1.109(1.009,1.219) |
|  | DM | 505074 | 904 | 3694405 | 0.24 | 1.746(1.596,1.91) | 1.248(1.139,1.366) | 1.185(1.079,1.301) |
| Esophagus (C15) | NFG | 936868 | 810 | 7223149 | 0.11 | 1(Ref.) | 1(Ref.) | 1(Ref.) |
|  | IFG | 513562 | 609 | 3896322 | 0.16 | 1.397(1.258,1.552) | 1.056(0.951,1.173) | 0.994(0.894,1.104) |
|  | DM | 505074 | 803 | 3694905 | 0.22 | 1.946(1.765,2.145) | 1.238(1.122,1.365) | 1.162(1.05,1.286) |
| Stomach (C16) | NFG | 936868 | 8781 | 7196113 | 1.22 | 1(Ref.) | 1(Ref.) | 1(Ref.) |
|  | IFG | 513562 | 6171 | 3877065 | 1.59 | 1.305(1.264,1.349) | 1.055(1.021,1.09) | 1.051(1.017,1.086) |
|  | DM | 505074 | 7881 | 3670887 | 2.15 | 1.76(1.708,1.815) | 1.238(1.201,1.277) | 1.234(1.195,1.274) |
| Colorectal (C18-C20) | NFG | 936868 | 12084 | 7187462 | 1.68 | 1(Ref.) | 1(Ref.) | 1(Ref.) |
|  | IFG | 513562 | 8610 | 3871251 | 2.22 | 1.325(1.289,1.362) | 1.11(1.08,1.142) | 1.093(1.063,1.124) |
|  | DM | 505074 | 10899 | 3664469 | 2.97 | 1.774(1.729,1.821) | 1.319(1.285,1.354) | 1.284(1.25,1.32) |
| Liver (C22) | NFG | 936868 | 5813 | 7213177 | 0.81 | 1(Ref.) | 1(Ref.) | 1(Ref.) |
|  | IFG | 513562 | 4555 | 3888362 | 1.17 | 1.456(1.401,1.514) | 1.184(1.139,1.231) | 1.18(1.135,1.227) |
|  | DM | 505074 | 7971 | 3681588 | 2.17 | 2.697(2.608,2.79) | 1.917(1.853,1.983) | 1.98(1.911,2.051) |
| Gallbladder and bile duct (C23-C24) | NFG | 936868 | 2911 | 7219810 | 0.40 | 1(Ref.) | 1(Ref.) | 1(Ref.) |
|  | IFG | 513562 | 2217 | 3893914 | 0.57 | 1.416(1.34,1.496) | 1.158(1.096,1.224) | 1.149(1.087,1.215) |
|  | DM | 505074 | 2857 | 3692072 | 0.77 | 1.928(1.831,2.031) | 1.381(1.311,1.455) | 1.369(1.297,1.444) |
| Pancreas (C25) | NFG | 936868 | 5885 | 7213033 | 0.82 | 1(Ref.) | 1(Ref.) | 1(Ref.) |
|  | IFG | 513562 | 4293 | 3888967 | 1.10 | 1.357(1.305,1.412) | 1.131(1.088,1.177) | 1.119(1.075,1.164) |
|  | DM | 505074 | 5989 | 3685450 | 1.63 | 2.006(1.935,2.079) | 1.479(1.426,1.533) | 1.433(1.381,1.488) |
| Larynx (C32) | NFG | 936868 | 389 | 7223788 | 0.05 | 1(Ref.) | 1(Ref.) | 1(Ref.) |
|  | IFG | 513562 | 300 | 3896708 | 0.08 | 1.43(1.23,1.663) | 1.062(0.913,1.234) | 1.02(0.877,1.187) |
|  | DM | 505074 | 398 | 3695415 | 0.11 | 2.004(1.742,2.304) | 1.239(1.077,1.426) | 1.163(1.007,1.344) |
| Lung (C33-C34) | NFG | 936868 | 10578 | 7204295 | 1.47 | 1(Ref.) | 1(Ref.) | 1(Ref.) |
|  | IFG | 513562 | 7373 | 3883874 | 1.90 | 1.296(1.258,1.335) | 1.02(0.99,1.051) | 1.018(0.988,1.049) |
|  | DM | 505074 | 9641 | 3680322 | 2.62 | 1.793(1.745,1.844) | 1.209(1.176,1.243) | 1.162(1.129,1.196) |
| Skin (C43-C44) | NFG | 936868 | 3189 | 7214514 | 0.44 | 1(Ref.) | 1(Ref.) | 1(Ref.) |
|  | IFG | 513562 | 1952 | 3891383 | 0.50 | 1.14(1.077,1.206) | 0.945(0.893,1) | 0.941(0.889,0.996) |
|  | DM | 505074 | 2314 | 3689337 | 0.63 | 1.432(1.357,1.511) | 1.051(0.996,1.109) | 1.034(0.978,1.093) |
| Kidney (C64) | NFG | 936868 | 2132 | 7218268 | 0.30 | 1(Ref.) | 1(Ref.) | 1(Ref.) |
|  | IFG | 513562 | 1510 | 3893012 | 0.39 | 1.317(1.233,1.407) | 1.1(1.029,1.175) | 1.035(0.969,1.107) |
|  | DM | 505074 | 1895 | 3690983 | 0.51 | 1.749(1.644,1.861) | 1.312(1.232,1.398) | 1.106(1.037,1.18) |
| Bladder (C67) | NFG | 936868 | 3065 | 7215442 | 0.42 | 1(Ref.) | 1(Ref.) | 1(Ref.) |
|  | IFG | 513562 | 2217 | 3890765 | 0.57 | 1.346(1.275,1.422) | 1.028(0.973,1.086) | 1.02(0.965,1.077) |
|  | DM | 505074 | 2831 | 3688424 | 0.77 | 1.819(1.728,1.914) | 1.173(1.114,1.234) | 1.114(1.056,1.174) |
| Brain and central nervous system (C70-C72) | NFG | 936868 | 1066 | 7222882 | 0.15 | 1(Ref.) | 1(Ref.) | 1(Ref.) |
|  | IFG | 513562 | 686 | 3896261 | 0.18 | 1.194(1.085,1.314) | 1.017(0.924,1.12) | 1.019(0.925,1.122) |
|  | DM | 505074 | 787 | 3695372 | 0.21 | 1.446(1.319,1.586) | 1.104(1.005,1.212) | 1.083(0.984,1.193) |
| Thyroid (C73) | NFG | 936868 | 6159 | 7200384 | 0.86 | 1(Ref.) | 1(Ref.) | 1(Ref.) |
|  | IFG | 513562 | 2869 | 3886273 | 0.74 | 0.863(0.826,0.902) | 1.076(1.029,1.126) | 1.064(1.017,1.114) |
|  | DM | 505074 | 2301 | 3687595 | 0.62 | 0.73(0.695,0.765) | 1.039(0.988,1.093) | 0.996(0.945,1.049) |
| Hodgkin's lymphoma (C81) | NFG | 936868 | 72 | 7224794 | 0.01 | 1(Ref.) | 1(Ref.) | 1(Ref.) |
|  | IFG | 513562 | 56 | 3897452 | 0.01 | 1.441(1.016,2.043) | 1.221(0.859,1.734) | 1.256(0.883,1.787) |
|  | DM | 505074 | 65 | 3696459 | 0.02 | 1.761(1.259,2.463) | 1.338(0.952,1.879) | 1.402(0.985,1.995) |
| Non- Hodgkin's lymphoma (C82-C86, C96) | NFG | 936868 | 1810 | 7220391 | 0.25 | 1(Ref.) | 1(Ref.) | 1(Ref.) |
|  | IFG | 513562 | 1129 | 3894944 | 0.29 | 1.158(1.075,1.247) | 0.976(0.906,1.052) | 0.986(0.915,1.063) |
|  | DM | 505074 | 1285 | 3693915 | 0.35 | 1.392(1.296,1.496) | 1.046(0.973,1.124) | 1.072(0.995,1.156) |
| Multiple myeloma (C90) | NFG | 936868 | 1309 | 7221589 | 0.18 | 1(Ref.) | 1(Ref.) | 1(Ref.) |
|  | IFG | 513562 | 791 | 3895721 | 0.20 | 1.122(1.027,1.225) | 0.925(0.847,1.011) | 0.931(0.852,1.018) |
|  | DM | 505074 | 1013 | 3694363 | 0.27 | 1.516(1.397,1.646) | 1.099(1.012,1.194) | 1.101(1.011,1.2) |
| Leukemia (C91-C95) | NFG | 936868 | 1174 | 7222645 | 0.16 | 1(Ref.) | 1(Ref.) | 1(Ref.) |
|  | IFG | 513562 | 699 | 3896329 | 0.18 | 1.105(1.006,1.213) | 0.931(0.848,1.023) | 0.939(0.854,1.032) |
|  | DM | 505074 | 802 | 3695212 | 0.22 | 1.338(1.223,1.464) | 1.007(0.919,1.102) | 1.029(0.937,1.131) |
| Prostate (C61) | NFG | 381404 | 7876 | 2891214 | 2.72 | 1(Ref.) | 1(Ref.) | 1(Ref.) |
|  | IFG | 260024 | 6055 | 1942188 | 3.12 | 1.15(1.112,1.189) | 1.037(1.002,1.072) | 1.018(0.984,1.053) |
|  | DM | 277265 | 6860 | 1994756 | 3.44 | 1.275(1.235,1.317) | 1.024(0.991,1.058) | 0.975(0.944,1.009) |
| Testis (C62) | NFG | 381404 | 96 | 2915636 | 0.03 | 1(Ref.) | 1(Ref.) | 1(Ref.) |
|  | IFG | 260024 | 51 | 1960034 | 0.03 | 0.787(0.56,1.105) | 0.735(0.523,1.033) | 0.731(0.519,1.029) |
|  | DM | 277265 | 59 | 2014681 | 0.03 | 0.88(0.636,1.217) | 0.76(0.549,1.053) | 0.738(0.527,1.034) |
| Breast (C50) | NFG | 555464 | 5839 | 4287421 | 1.36 | 1(Ref.) | 1(Ref.) | 1(Ref.) |
|  | IFG | 253538 | 2736 | 1927270 | 1.42 | 1.044(0.997,1.092) | 1.138(1.086,1.192) | 1.127(1.075,1.181) |
|  | DM | 227809 | 2146 | 1673974 | 1.28 | 0.944(0.898,0.992) | 1.086(1.032,1.144) | 1.051(0.996,1.109) |
| Cervix (C53) | NFG | 555464 | 1094 | 4305089 | 0.25 | 1(Ref.) | 1(Ref.) | 1(Ref.) |
|  | IFG | 253538 | 522 | 1935679 | 0.27 | 1.061(0.956,1.177) | 1.042(0.937,1.158) | 1.037(0.933,1.154) |
|  | DM | 227809 | 494 | 1680244 | 0.29 | 1.156(1.04,1.286) | 1.123(1.006,1.254) | 1.125(1.003,1.262) |
| Uterus (C54) | NFG | 555464 | 872 | 4305978 | 0.20 | 1(Ref.) | 1(Ref.) | 1(Ref.) |
|  | IFG | 253538 | 424 | 1935860 | 0.22 | 1.082(0.964,1.215) | 1.222(1.085,1.376) | 1.188(1.054,1.338) |
|  | DM | 227809 | 385 | 1680383 | 0.23 | 1.134(1.006,1.278) | 1.382(1.218,1.569) | 1.245(1.092,1.421) |
| Ovary (C56) | NFG | 555464 | 1831 | 4304235 | 0.43 | 1(Ref.) | 1(Ref.) | 1(Ref.) |
|  | IFG | 253538 | 876 | 1935287 | 0.45 | 1.068(0.985,1.157) | 1.048(0.966,1.137) | 1.036(0.955,1.125) |
|  | DM | 227809 | 833 | 1679911 | 0.50 | 1.175(1.083,1.276) | 1.14(1.047,1.241) | 1.104(1.011,1.206) |

^a^ Model 1, Non-adjusted. ^b^ Model 2, adjusted for age and sex. ^c^ Model 3, Adjusted for age, sex, smoking, alcohol drinking, regular exercise, low income, previous history of hypertension and dyslipidemia. Abbreviations: DM, diabetes mellitus; BMI, body mass index; WC, waist circumference; HR, hazard ratio; CI, confidential interval; NGF, normal fasting glucose; IFG, impaired fasting glucose.

**Table S4.** Incidence rates and hazard ratios of site-specific cancers by body mass index status

| Cancer site | BMI group, kg/m^2^ | Number | Cancer | Follow-up Duration, Person-year | Incidence Rate, Per 1000 person-year | Model 1, HR (95% CI) ^a^ | Model 2, HR (95% CI) ^b^ | Model 3, HR (95% CI) ^c^ |
| --- | --- | --- | --- | --- | --- | --- | --- | --- |
| Lips, oral cavity and pharynx (C00-C14) | < 18.5 | 62179 | 84 | 432705 | 0.19 | 1.054(0.842,1.319) | 1.331(1.063,1.666) | 1.31(1.046,1.641) |
|  | 18.5–23 | 596875 | 825 | 4456762 | 0.19 | 1(Ref.) | 1(Ref.) | 1(Ref.) |
|  | 23–25 | 469846 | 684 | 3583757 | 0.19 | 1.029(0.93,1.139) | 0.883(0.798,0.978) | 0.885(0.799,0.98) |
|  | 25–30 | 700046 | 970 | 5373248 | 0.18 | 0.973(0.887,1.068) | 0.85(0.774,0.932) | 0.838(0.762,0.922) |
|  | ≥ 30 | 126558 | 126 | 965679 | 0.13 | 0.705(0.585,0.851) | 0.809(0.67,0.977) | 0.766(0.632,0.927) |
| Esophagus (C15) | < 18.5 | 62179 | 101 | 432707 | 0.23 | 1.378(1.12,1.696) | 1.804(1.466,2.221) | 1.757(1.426,2.166) |
|  | 18.5–23 | 596875 | 758 | 4457222 | 0.17 | 1(Ref.) | 1(Ref.) | 1(Ref.) |
|  | 23–25 | 469846 | 575 | 3584351 | 0.16 | 0.942(0.845,1.05) | 0.782(0.701,0.871) | 0.791(0.709,0.882) |
|  | 25–30 | 700046 | 706 | 5374276 | 0.13 | 0.771(0.696,0.855) | 0.668(0.603,0.741) | 0.67(0.603,0.744) |
|  | ≥ 30 | 126558 | 82 | 965820 | 0.08 | 0.499(0.398,0.627) | 0.667(0.53,0.839) | 0.65(0.515,0.819) |
| Stomach (C16) | < 18.5 | 62179 | 452 | 431612 | 1.05 | 0.736(0.669,0.809) | 0.902(0.82,0.992) | 0.89(0.809,0.979) |
|  | 18.5–23 | 596875 | 6333 | 4439037 | 1.43 | 1(Ref.) | 1(Ref.) | 1(Ref.) |
|  | 23–25 | 469846 | 5895 | 3565965 | 1.65 | 1.159(1.118,1.201) | 1.012(0.977,1.049) | 1.018(0.983,1.055) |
|  | 25–30 | 700046 | 8980 | 5345300 | 1.68 | 1.178(1.14,1.216) | 1.063(1.029,1.098) | 1.07(1.035,1.106) |
|  | ≥ 30 | 126558 | 1173 | 962150 | 1.22 | 0.855(0.803,0.91) | 1.048(0.984,1.116) | 1.052(0.987,1.121) |
| Colon (C18-C20) | < 18.5 | 62179 | 670 | 431166 | 1.55 | 0.795(0.735,0.86) | 0.952(0.88,1.029) | 0.951(0.879,1.029) |
|  | 18.5–23 | 596875 | 8695 | 4433159 | 1.96 | 1(Ref.) | 1(Ref.) | 1(Ref.) |
|  | 23–25 | 469846 | 7990 | 3561395 | 2.24 | 1.143(1.109,1.178) | 1.025(0.995,1.057) | 1.02(0.99,1.052) |
|  | 25–30 | 700046 | 12301 | 5337457 | 2.30 | 1.174(1.142,1.207) | 1.083(1.054,1.113) | 1.067(1.038,1.097) |
|  | ≥ 30 | 126558 | 1937 | 960005 | 2.02 | 1.029(0.979,1.081) | 1.215(1.157,1.277) | 1.175(1.117,1.235) |
| Liver (C22) | < 18.5 | 62179 | 414 | 432160 | 0.96 | 0.872(0.789,0.964) | 1.077(0.974,1.19) | 1.03(0.931,1.139) |
|  | 18.5–23 | 596875 | 4909 | 4449705 | 1.10 | 1(Ref.) | 1(Ref.) | 1(Ref.) |
|  | 23–25 | 469846 | 4509 | 3576363 | 1.26 | 1.141(1.096,1.188) | 0.997(0.957,1.038) | 1.015(0.974,1.057) |
|  | 25–30 | 700046 | 7213 | 5361423 | 1.35 | 1.217(1.174,1.262) | 1.099(1.059,1.139) | 1.116(1.075,1.158) |
|  | ≥ 30 | 126558 | 1294 | 963475 | 1.34 | 1.217(1.145,1.294) | 1.496(1.406,1.591) | 1.483(1.393,1.58) |
| Gallbladder and bile duct (C23-C24) | < 18.5 | 62179 | 176 | 432658 | 0.41 | 0.873(0.748,1.018) | 1(0.857,1.166) | 1(0.857,1.166) |
|  | 18.5–23 | 596875 | 2088 | 4455545 | 0.47 | 1(Ref.) | 1(Ref.) | 1(Ref.) |
|  | 23–25 | 469846 | 2029 | 3582093 | 0.57 | 1.207(1.135,1.283) | 1.106(1.04,1.175) | 1.103(1.037,1.173) |
|  | 25–30 | 700046 | 3198 | 5370322 | 0.60 | 1.268(1.2,1.34) | 1.221(1.155,1.29) | 1.214(1.147,1.284) |
|  | ≥ 30 | 126558 | 494 | 965178 | 0.51 | 1.092(0.99,1.204) | 1.421(1.288,1.569) | 1.404(1.27,1.551) |
| Pancreas (C25) | < 18.5 | 62179 | 335 | 432321 | 0.77 | 0.791(0.708,0.884) | 0.949(0.849,1.06) | 0.952(0.852,1.064) |
|  | 18.5–23 | 596875 | 4398 | 4450649 | 0.99 | 1(Ref.) | 1(Ref.) | 1(Ref.) |
|  | 23–25 | 469846 | 4176 | 3577300 | 1.17 | 1.178(1.129,1.229) | 1.059(1.015,1.104) | 1.051(1.007,1.097) |
|  | 25–30 | 700046 | 6321 | 5362999 | 1.18 | 1.189(1.144,1.236) | 1.103(1.061,1.146) | 1.082(1.041,1.126) |
|  | ≥ 30 | 126558 | 937 | 964182 | 0.97 | 0.983(0.916,1.055) | 1.183(1.102,1.269) | 1.131(1.053,1.216) |
| Larynx (C32) | < 18.5 | 62179 | 57 | 432743 | 0.13 | 1.57(1.188,2.075) | 2.092(1.582,2.764) | 1.929(1.457,2.554) |
|  | 18.5–23 | 596875 | 375 | 4457720 | 0.08 | 1(Ref.) | 1(Ref.) | 1(Ref.) |
|  | 23–25 | 469846 | 278 | 3584703 | 0.08 | 0.921(0.789,1.076) | 0.754(0.646,0.881) | 0.787(0.673,0.92) |
|  | 25–30 | 700046 | 345 | 5374828 | 0.06 | 0.762(0.659,0.882) | 0.654(0.565,0.758) | 0.687(0.591,0.798) |
|  | ≥ 30 | 126558 | 32 | 965917 | 0.03 | 0.394(0.274,0.565) | 0.542(0.377,0.78) | 0.552(0.383,0.797) |
| Lung(C33-C34) | < 18.5 | 62179 | 839 | 431757 | 1.94 | 0.986(0.919,1.058) | 1.214(1.131,1.303) | 1.139(1.061,1.223) |
|  | 18.5–23 | 596875 | 8802 | 4443263 | 1.98 | 1(Ref.) | 1(Ref.) | 1(Ref.) |
|  | 23–25 | 469846 | 7114 | 3572450 | 1.99 | 1.003(0.973,1.035) | 0.875(0.848,0.903) | 0.901(0.873,0.929) |
|  | 25–30 | 700046 | 9673 | 5357297 | 1.81 | 0.91(0.884,0.936) | 0.833(0.809,0.857) | 0.862(0.837,0.888) |
|  | ≥ 30 | 126558 | 1164 | 963724 | 1.21 | 0.609(0.573,0.648) | 0.811(0.763,0.862) | 0.821(0.771,0.874) |
| Skin (C43-C44) | < 18.5 | 62179 | 203 | 432253 | 0.47 | 0.9(0.78,1.039) | 0.999(0.866,1.154) | 1.021(0.884,1.179) |
|  | 18.5–23 | 596875 | 2348 | 4451262 | 0.53 | 1(Ref.) | 1(Ref.) | 1(Ref.) |
|  | 23–25 | 469846 | 1945 | 3579293 | 0.54 | 1.026(0.966,1.09) | 0.963(0.907,1.023) | 0.95(0.895,1.01) |
|  | 25–30 | 700046 | 2556 | 5367623 | 0.48 | 0.899(0.85,0.951) | 0.884(0.836,0.936) | 0.863(0.815,0.913) |
|  | ≥ 30 | 126558 | 403 | 964804 | 0.42 | 0.792(0.713,0.88) | 1.013(0.911,1.126) | 0.974(0.875,1.084) |
| Kidney (C64) | < 18.5 | 62179 | 70 | 432676 | 0.16 | 0.601(0.473,0.765) | 0.755(0.593,0.96) | 0.818(0.642,1.041) |
|  | 18.5–23 | 596875 | 1206 | 4455209 | 0.27 | 1(Ref.) | 1(Ref.) | 1(Ref.) |
|  | 23–25 | 469846 | 1364 | 3581451 | 0.38 | 1.404(1.299,1.517) | 1.207(1.117,1.305) | 1.134(1.049,1.226) |
|  | 25–30 | 700046 | 2432 | 5368339 | 0.45 | 1.67(1.558,1.789) | 1.44(1.343,1.543) | 1.272(1.185,1.364) |
|  | ≥ 30 | 126558 | 465 | 964588 | 0.48 | 1.781(1.6,1.982) | 1.908(1.713,2.125) | 1.528(1.369,1.706) |
| Bladder (C67) | < 18.5 | 62179 | 166 | 432458 | 0.38 | 0.766(0.654,0.897) | 0.962(0.821,1.126) | 0.957(0.817,1.121) |
|  | 18.5–23 | 596875 | 2247 | 4452116 | 0.50 | 1(Ref.) | 1(Ref.) | 1(Ref.) |
|  | 23–25 | 469846 | 2167 | 3578873 | 0.61 | 1.197(1.128,1.269) | 1.025(0.966,1.087) | 1.023(0.964,1.086) |
|  | 25–30 | 700046 | 3180 | 5366177 | 0.59 | 1.171(1.109,1.236) | 1.064(1.008,1.123) | 1.055(0.998,1.115) |
|  | ≥ 30 | 126558 | 353 | 965007 | 0.37 | 0.725(0.648,0.811) | 1.033(0.922,1.156) | 1.002(0.894,1.123) |
| Brain and central nervous system (C70-C72) | < 18.5 | 62179 | 49 | 432781 | 0.11 | 0.714(0.535,0.953) | 0.846(0.634,1.131) | 0.841(0.629,1.124) |
|  | 18.5–23 | 596875 | 710 | 4457433 | 0.16 | 1(Ref.) | 1(Ref.) | 1(Ref.) |
|  | 23–25 | 469846 | 658 | 3584364 | 0.18 | 1.152(1.036,1.281) | 1.043(0.938,1.16) | 1.046(0.94,1.164) |
|  | 25–30 | 700046 | 982 | 5374156 | 0.18 | 1.146(1.041,1.263) | 1.062(0.964,1.17) | 1.066(0.966,1.176) |
|  | ≥ 30 | 126558 | 140 | 965781 | 0.14 | 0.91(0.759,1.09) | 1.044(0.87,1.252) | 1.038(0.863,1.249) |
| Thyroid (C73) | < 18.5 | 62179 | 301 | 431703 | 0.70 | 0.926(0.823,1.042) | 0.666(0.592,0.75) | 0.667(0.592,0.751) |
|  | 18.5–23 | 596875 | 3346 | 4445976 | 0.75 | 1(Ref.) | 1(Ref.) | 1(Ref.) |
|  | 23–25 | 469846 | 2510 | 3575468 | 0.70 | 0.933(0.886,0.982) | 1.178(1.118,1.242) | 1.168(1.108,1.231) |
|  | 25–30 | 700046 | 4228 | 5358777 | 0.79 | 1.049(1.002,1.097) | 1.374(1.311,1.44) | 1.346(1.284,1.412) |
|  | ≥ 30 | 126558 | 944 | 962328 | 0.98 | 1.304(1.214,1.402) | 1.436(1.336,1.544) | 1.387(1.288,1.495) |
| Hodgkin's lymphoma (C81) | < 18.5 | 62179 | 4 | 432865 | 0.01 | 0.723(0.262,1.992) | 0.869(0.315,2.396) | 0.843(0.305,2.327) |
|  | 18.5–23 | 596875 | 57 | 4458638 | 0.01 | 1(Ref.) | 1(Ref.) | 1(Ref.) |
|  | 23–25 | 469846 | 52 | 3585468 | 0.01 | 1.134(0.779,1.652) | 1.01(0.693,1.472) | 1.034(0.708,1.509) |
|  | 25–30 | 700046 | 66 | 5375755 | 0.01 | 0.96(0.674,1.369) | 0.871(0.611,1.243) | 0.904(0.63,1.298) |
|  | ≥ 30 | 126558 | 14 | 965979 | 0.01 | 1.133(0.632,2.034) | 1.284(0.714,2.311) | 1.341(0.737,2.44) |
| Non- Hodgkin's lymphoma (C82-C86, C96) | < 18.5 | 62179 | 74 | 432716 | 0.17 | 0.659(0.521,0.834) | 0.793(0.627,1.003) | 0.781(0.617,0.988) |
|  | 18.5–23 | 596875 | 1160 | 4455973 | 0.26 | 1(Ref.) | 1(Ref.) | 1(Ref.) |
|  | 23–25 | 469846 | 1067 | 3583084 | 0.30 | 1.142(1.051,1.241) | 1.024(0.942,1.113) | 1.034(0.951,1.125) |
|  | 25–30 | 700046 | 1654 | 5372100 | 0.31 | 1.181(1.096,1.273) | 1.084(1.005,1.168) | 1.104(1.023,1.192) |
|  | ≥ 30 | 126558 | 269 | 965377 | 0.28 | 1.07(0.937,1.222) | 1.235(1.081,1.411) | 1.27(1.11,1.455) |
| Multiple myeloma (C90) | < 18.5 | 62179 | 59 | 432789 | 0.14 | 0.703(0.54,0.915) | 0.845(0.649,1.1) | 0.85(0.653,1.107) |
|  | 18.5–23 | 596875 | 869 | 4456625 | 0.19 | 1(Ref.) | 1(Ref.) | 1(Ref.) |
|  | 23–25 | 469846 | 812 | 3583609 | 0.23 | 1.161(1.055,1.277) | 1.033(0.939,1.137) | 1.029(0.935,1.133) |
|  | 25–30 | 700046 | 1209 | 5373020 | 0.23 | 1.153(1.056,1.258) | 1.058(0.97,1.155) | 1.052(0.962,1.15) |
|  | ≥ 30 | 126558 | 164 | 965631 | 0.17 | 0.872(0.738,1.03) | 1.049(0.887,1.24) | 1.038(0.876,1.231) |
| Leukemia (C91-C95) | < 18.5 | 62179 | 49 | 432797 | 0.11 | 0.666(0.499,0.889) | 0.803(0.601,1.071) | 0.791(0.592,1.057) |
|  | 18.5–23 | 596875 | 760 | 4457350 | 0.17 | 1(Ref.) | 1(Ref.) | 1(Ref.) |
|  | 23–25 | 469846 | 661 | 3584349 | 0.18 | 1.08(0.973,1.199) | 0.966(0.87,1.072) | 0.972(0.876,1.08) |
|  | 25–30 | 700046 | 1030 | 5374027 | 0.19 | 1.123(1.022,1.233) | 1.024(0.933,1.125) | 1.036(0.942,1.141) |
|  | ≥ 30 | 126558 | 175 | 965662 | 0.18 | 1.063(0.902,1.253) | 1.216(1.031,1.434) | 1.234(1.043,1.459) |
| Prostate (C61) | < 18.5 | 18300 | 290 | 117503 | 2.47 | 0.805(0.715,0.906) | 0.76(0.675,0.855) | 0.787(0.699,0.886) |
|  | 18.5–23 | 235467 | 5300 | 1699323 | 3.12 | 1(Ref.) | 1(Ref.) | 1(Ref.) |
|  | 23–25 | 234928 | 5931 | 1757059 | 3.38 | 1.077(1.037,1.117) | 1.14(1.099,1.184) | 1.118(1.077,1.161) |
|  | 25–30 | 370125 | 8455 | 2801150 | 3.02 | 0.962(0.93,0.996) | 1.167(1.127,1.208) | 1.129(1.09,1.169) |
|  | ≥ 30 | 59873 | 815 | 453122 | 1.80 | 0.576(0.535,0.62) | 1.132(1.05,1.219) | 1.078(1,1.163) |
| Testis (C62) | < 18.5 | 18300 | 3 | 118212 | 0.03 | 1.016(0.315,3.277) | 1.003(0.311,3.235) | 1.017(0.314,3.288) |
|  | 18.5–23 | 235467 | 42 | 1714646 | 0.02 | 1(Ref.) | 1(Ref.) | 1(Ref.) |
|  | 23–25 | 234928 | 56 | 1775201 | 0.03 | 1.296(0.869,1.934) | 1.314(0.88,1.96) | 1.306(0.874,1.953) |
|  | 25–30 | 370125 | 98 | 2826740 | 0.03 | 1.426(0.993,2.047) | 1.536(1.069,2.208) | 1.527(1.055,2.209) |
|  | ≥ 30 | 59873 | 7 | 455551 | 0.02 | 0.63(0.283,1.403) | 0.858(0.383,1.925) | 0.854(0.378,1.931) |
| Breast (C50) | < 18.5 | 43879 | 299 | 313653 | 0.95 | 0.778(0.691,0.876) | 0.672(0.596,0.756) | 0.682(0.605,0.768) |
|  | 18.5–23 | 361408 | 3362 | 2731467 | 1.23 | 1(Ref.) | 1(Ref.) | 1(Ref.) |
|  | 23–25 | 234918 | 2446 | 1801167 | 1.36 | 1.102(1.046,1.161) | 1.213(1.151,1.279) | 1.207(1.145,1.273) |
|  | 25–30 | 329921 | 3726 | 2535158 | 1.47 | 1.193(1.138,1.25) | 1.342(1.279,1.408) | 1.338(1.274,1.405) |
|  | ≥ 30 | 66685 | 888 | 507221 | 1.75 | 1.423(1.322,1.532) | 1.514(1.406,1.631) | 1.518(1.407,1.638) |
| Cervix (C53) | < 18.5 | 43879 | 76 | 314449 | 0.24 | 0.969(0.765,1.228) | 0.996(0.785,1.264) | 0.994(0.783,1.261) |
|  | 18.5–23 | 361408 | 683 | 2741632 | 0.25 | 1(Ref.) | 1(Ref.) | 1(Ref.) |
|  | 23–25 | 234918 | 475 | 1808550 | 0.26 | 1.055(0.938,1.186) | 1.036(0.921,1.167) | 1.04(0.923,1.171) |
|  | 25–30 | 329921 | 714 | 2546460 | 0.28 | 1.126(1.014,1.251) | 1.102(0.991,1.226) | 1.105(0.991,1.232) |
|  | ≥ 30 | 66685 | 162 | 509922 | 0.32 | 1.275(1.074,1.513) | 1.261(1.062,1.497) | 1.256(1.054,1.496) |
| Uterus (C54) | < 18.5 | 43879 | 25 | 314578 | 0.08 | 0.487(0.326,0.729) | 0.399(0.266,0.598) | 0.405(0.27,0.606) |
|  | 18.5–23 | 361408 | 448 | 2742535 | 0.16 | 1(Ref.) | 1(Ref.) | 1(Ref.) |
|  | 23–25 | 234918 | 362 | 1808896 | 0.20 | 1.225(1.066,1.406) | 1.401(1.217,1.613) | 1.374(1.193,1.582) |
|  | 25–30 | 329921 | 582 | 2546707 | 0.23 | 1.398(1.236,1.581) | 1.649(1.452,1.872) | 1.596(1.402,1.816) |
|  | ≥ 30 | 66685 | 264 | 509506 | 0.52 | 3.171(2.724,3.692) | 3.465(2.973,4.038) | 3.284(2.802,3.849) |
| Ovary (C56) | < 18.5 | 43879 | 83 | 314460 | 0.26 | 0.663(0.531,0.829) | 0.678(0.542,0.848) | 0.681(0.544,0.852) |
|  | 18.5–23 | 361408 | 1103 | 2741243 | 0.40 | 1(Ref.) | 1(Ref.) | 1(Ref.) |
|  | 23–25 | 234918 | 799 | 1808226 | 0.44 | 1.094(0.998,1.198) | 1.079(0.984,1.183) | 1.072(0.978,1.176) |
|  | 25–30 | 329921 | 1220 | 2545859 | 0.48 | 1.185(1.092,1.286) | 1.165(1.073,1.266) | 1.151(1.058,1.253) |
|  | ≥ 30 | 66685 | 335 | 509644 | 0.66 | 1.633(1.445,1.846) | 1.619(1.433,1.83) | 1.584(1.397,1.796) |

^a^ Model 1, Non-adjusted. ^b^ Model 2, adjusted for age and sex. ^c^ Model 3, Adjusted for age, sex, smoking, alcohol drinking, regular exercise, low income, previous history of hypertension and dyslipidemia. Abbreviations: DM, diabetes mellitus; BMI, body mass index; WC, waist circumference; HR, hazard ratio; CI, confidential interval; NGF, normal fasting glucose; IFG, impaired fasting glucose.

**Table S5.** Incidence rates and hazard ratios of site-specific cancers by waist circumference status

| Cancer site | WC group, cm, (male/ female) | Number | Cancer | Follow-up Duration, Person-year | Incidence Rate, Per 1000 person-year | Model 1, HR (95% CI) ^a^ | Model 2, HR (95% CI) ^b^ | Model 3, HR (95% CI) ^c^ |
| --- | --- | --- | --- | --- | --- | --- | --- | --- |
| Lips, oral cavity and pharynx (C00-C14) | < 80/<75 | 476365 | 535 | 3600989 | 0.15 | 0.754(0.672,0.845) | 1.065(0.95,1.195) | 1.089(0.97,1.223) |
|  | 80–85/75–80 | 401285 | 567 | 3054060 | 0.19 | 0.941(0.841,1.053) | 0.992(0.887,1.11) | 1.002(0.895,1.121) |
|  | 85–90/80–85 | 436958 | 656 | 3325588 | 0.20 | 1(Ref.) | 1(Ref.) | 1(Ref.) |
|  | 90–95/85–90 | 324680 | 490 | 2460778 | 0.20 | 1.01(0.898,1.135) | 0.975(0.867,1.096) | 0.962(0.856,1.082) |
|  | 95–100/90–95 | 181355 | 251 | 1366008 | 0.18 | 0.933(0.806,1.079) | 0.941(0.814,1.089) | 0.917(0.793,1.061) |
|  | ≥ 100/ ≥ 95 | 134861 | 190 | 1004727 | 0.19 | 0.961(0.818,1.13) | 1.082(0.92,1.271) | 1.035(0.88,1.218) |
| Esophagus (C15) | < 80/<75 | 476365 | 485 | 3601416 | 0.13 | 0.871(0.769,0.986) | 1.334(1.178,1.51) | 1.358(1.198,1.54) |
|  | 80–85/75–80 | 401285 | 493 | 3054441 | 0.16 | 1.042(0.921,1.18) | 1.102(0.974,1.246) | 1.112(0.983,1.258) |
|  | 85–90/80–85 | 436958 | 515 | 3326211 | 0.15 | 1(Ref.) | 1(Ref.) | 1(Ref.) |
|  | 90–95/85–90 | 324680 | 385 | 2461244 | 0.16 | 1.011(0.886,1.153) | 0.965(0.846,1.101) | 0.952(0.834,1.087) |
|  | 95–100/90–95 | 181355 | 215 | 1366109 | 0.16 | 1.018(0.868,1.193) | 1.054(0.899,1.236) | 1.025(0.874,1.203) |
|  | ≥ 100/ ≥ 95 | 134861 | 129 | 1004954 | 0.13 | 0.831(0.685,1.008) | 1.021(0.841,1.238) | 0.981(0.808,1.191) |
| Stomach (C16) | < 80/<75 | 476365 | 3947 | 3589788 | 1.10 | 0.658(0.632,0.686) | 0.939(0.902,0.979) | 0.939(0.901,0.979) |
|  | 80–85/75–80 | 401285 | 4781 | 3039841 | 1.57 | 0.941(0.905,0.978) | 1.003(0.965,1.042) | 1.003(0.965,1.043) |
|  | 85–90/80–85 | 436958 | 5532 | 3308796 | 1.67 | 1(Ref.) | 1(Ref.) | 1(Ref.) |
|  | 90–95/85–90 | 324680 | 4567 | 2447023 | 1.87 | 1.116(1.073,1.161) | 1.072(1.031,1.115) | 1.071(1.03,1.114) |
|  | 95–100/90–95 | 181355 | 2438 | 1358451 | 1.79 | 1.073(1.024,1.126) | 1.064(1.015,1.116) | 1.062(1.012,1.114) |
|  | ≥ 100/ ≥ 95 | 134861 | 1568 | 1000166 | 1.57 | 0.938(0.887,0.992) | 1.037(0.981,1.097) | 1.033(0.976,1.093) |
| Colon (C18-C20) | < 80/<75 | 476365 | 5528 | 3585702 | 1.54 | 0.669(0.646,0.692) | 0.918(0.887,0.951) | 0.936(0.904,0.97) |
|  | 80–85/75–80 | 401285 | 6217 | 3036703 | 2.05 | 0.887(0.858,0.918) | 0.95(0.918,0.982) | 0.957(0.925,0.99) |
|  | 85–90/80–85 | 436958 | 7623 | 3303855 | 2.31 | 1(Ref.) | 1(Ref.) | 1(Ref.) |
|  | 90–95/85–90 | 324680 | 6157 | 2443246 | 2.52 | 1.092(1.056,1.13) | 1.052(1.017,1.088) | 1.044(1.01,1.08) |
|  | 95–100/90–95 | 181355 | 3554 | 1356010 | 2.62 | 1.137(1.092,1.183) | 1.103(1.06,1.148) | 1.087(1.045,1.132) |
|  | ≥ 100/ ≥ 95 | 134861 | 2514 | 997667 | 2.52 | 1.094(1.046,1.144) | 1.152(1.102,1.206) | 1.126(1.076,1.179) |
| Liver (C22) | < 80/<75 | 476365 | 3042 | 3596349 | 0.85 | 0.657(0.627,0.688) | 0.94(0.897,0.985) | 0.929(0.887,0.974) |
|  | 80–85/75–80 | 401285 | 3464 | 3048570 | 1.14 | 0.882(0.843,0.922) | 0.941(0.899,0.984) | 0.94(0.899,0.983) |
|  | 85–90/80–85 | 436958 | 4277 | 3318888 | 1.29 | 1(Ref.) | 1(Ref.) | 1(Ref.) |
|  | 90–95/85–90 | 324680 | 3575 | 2455042 | 1.46 | 1.13(1.081,1.181) | 1.086(1.038,1.135) | 1.081(1.034,1.13) |
|  | 95–100/90–95 | 181355 | 2192 | 1362478 | 1.61 | 1.249(1.187,1.315) | 1.239(1.176,1.304) | 1.229(1.167,1.294) |
|  | ≥ 100/ ≥ 95 | 134861 | 1789 | 1001799 | 1.79 | 1.39(1.316,1.469) | 1.535(1.452,1.622) | 1.506(1.424,1.592) |
| Gallbladder and bile duct (C23-C24) | < 80/<75 | 476365 | 1281 | 3600272 | 0.36 | 0.611(0.569,0.656) | 0.878(0.818,0.942) | 0.887(0.826,0.953) |
|  | 80–85/75–80 | 401285 | 1529 | 3052940 | 0.50 | 0.859(0.803,0.919) | 0.941(0.88,1.006) | 0.944(0.883,1.01) |
|  | 85–90/80–85 | 436958 | 1938 | 3323967 | 0.58 | 1(Ref.) | 1(Ref.) | 1(Ref.) |
|  | 90–95/85–90 | 324680 | 1593 | 2459426 | 0.65 | 1.111(1.04,1.188) | 1.057(0.989,1.13) | 1.054(0.986,1.126) |
|  | 95–100/90–95 | 181355 | 975 | 1365050 | 0.71 | 1.227(1.136,1.325) | 1.153(1.067,1.245) | 1.146(1.061,1.238) |
|  | ≥ 100/ ≥ 95 | 134861 | 669 | 1004142 | 0.67 | 1.146(1.05,1.251) | 1.173(1.074,1.281) | 1.162(1.064,1.27) |
| Pancreas (C25) | < 80/<75 | 476365 | 2708 | 3596963 | 0.75 | 0.639(0.609,0.671) | 0.89(0.848,0.935) | 0.911(0.867,0.958) |
|  | 80–85/75–80 | 401285 | 3276 | 3049154 | 1.07 | 0.91(0.869,0.954) | 0.98(0.936,1.027) | 0.989(0.944,1.036) |
|  | 85–90/80–85 | 436958 | 3917 | 3319351 | 1.18 | 1(Ref.) | 1(Ref.) | 1(Ref.) |
|  | 90–95/85–90 | 324680 | 3126 | 2455988 | 1.27 | 1.079(1.03,1.131) | 1.036(0.989,1.086) | 1.027(0.98,1.077) |
|  | 95–100/90–95 | 181355 | 1853 | 1363095 | 1.36 | 1.154(1.092,1.22) | 1.112(1.052,1.175) | 1.093(1.034,1.156) |
|  | ≥ 100/ ≥ 95 | 134861 | 1287 | 1002899 | 1.28 | 1.092(1.025,1.163) | 1.143(1.073,1.218) | 1.112(1.044,1.185) |
| Larynx (C32) | < 80/<75 | 476365 | 237 | 3601699 | 0.07 | 0.901(0.754,1.078) | 1.403(1.173,1.678) | 1.38(1.151,1.654) |
|  | 80–85/75–80 | 401285 | 264 | 3054777 | 0.09 | 1.183(0.994,1.408) | 1.246(1.047,1.483) | 1.246(1.046,1.483) |
|  | 85–90/80–85 | 436958 | 243 | 3326593 | 0.07 | 1(Ref.) | 1(Ref.) | 1(Ref.) |
|  | 90–95/85–90 | 324680 | 181 | 2461518 | 0.07 | 1.007(0.831,1.22) | 0.96(0.792,1.164) | 0.949(0.783,1.151) |
|  | 95–100/90–95 | 181355 | 113 | 1366244 | 0.08 | 1.133(0.906,1.416) | 1.189(0.951,1.486) | 1.16(0.927,1.451) |
|  | ≥ 100/ ≥ 95 | 134861 | 49 | 1005080 | 0.05 | 0.668(0.491,0.908) | 0.848(0.624,1.153) | 0.815(0.599,1.109) |
| Lung(C33-C34) | < 80/<75 | 476365 | 5288 | 3592938 | 1.47 | 0.739(0.712,0.766) | 1.095(1.056,1.135) | 1.079(1.04,1.119) |
|  | 80–85/75–80 | 401285 | 5728 | 3044980 | 1.88 | 0.943(0.911,0.977) | 1.014(0.979,1.051) | 1.011(0.976,1.048) |
|  | 85–90/80–85 | 436958 | 6610 | 3314931 | 1.99 | 1(Ref.) | 1(Ref.) | 1(Ref.) |
|  | 90–95/85–90 | 324680 | 5217 | 2452553 | 2.13 | 1.067(1.029,1.107) | 1.017(0.981,1.055) | 1.012(0.976,1.049) |
|  | 95–100/90–95 | 181355 | 2831 | 1361471 | 2.08 | 1.044(0.999,1.091) | 1.031(0.986,1.077) | 1.017(0.973,1.063) |
|  | ≥ 100/ ≥ 95 | 134861 | 1918 | 1001618 | 1.91 | 0.963(0.915,1.013) | 1.08(1.027,1.137) | 1.055(1.003,1.11) |
| Skin (C43-C44) | < 80/<75 | 476365 | 1363 | 3598007 | 0.38 | 0.737(0.687,0.792) | 1.057(0.985,1.135) | 1.075(1,1.155) |
|  | 80–85/75–80 | 401285 | 1513 | 3050768 | 0.50 | 0.963(0.899,1.032) | 1.072(1.001,1.149) | 1.079(1.007,1.157) |
|  | 85–90/80–85 | 436958 | 1711 | 3321640 | 0.52 | 1(Ref.) | 1(Ref.) | 1(Ref.) |
|  | 90–95/85–90 | 324680 | 1469 | 2457369 | 0.60 | 1.162(1.083,1.246) | 1.1(1.026,1.18) | 1.095(1.021,1.174) |
|  | 95–100/90–95 | 181355 | 805 | 1364056 | 0.59 | 1.149(1.057,1.249) | 1.043(0.959,1.135) | 1.034(0.951,1.125) |
|  | ≥ 100/ ≥ 95 | 134861 | 594 | 1003396 | 0.59 | 1.157(1.054,1.27) | 1.119(1.019,1.228) | 1.103(1.004,1.212) |
| Kidney (C64) | < 80/<75 | 476365 | 798 | 3599945 | 0.22 | 0.564(0.517,0.616) | 0.759(0.695,0.83) | 0.858(0.785,0.938) |
|  | 80–85/75–80 | 401285 | 1025 | 3052507 | 0.34 | 0.854(0.787,0.926) | 0.887(0.817,0.962) | 0.928(0.855,1.007) |
|  | 85–90/80–85 | 436958 | 1307 | 3323273 | 0.39 | 1(Ref.) | 1(Ref.) | 1(Ref.) |
|  | 90–95/85–90 | 324680 | 1218 | 2458261 | 0.50 | 1.261(1.166,1.363) | 1.227(1.135,1.327) | 1.182(1.093,1.278) |
|  | 95–100/90–95 | 181355 | 647 | 1364650 | 0.47 | 1.208(1.099,1.327) | 1.232(1.122,1.354) | 1.15(1.046,1.264) |
|  | ≥ 100/ ≥ 95 | 134861 | 542 | 1003626 | 0.54 | 1.378(1.247,1.524) | 1.538(1.391,1.7) | 1.374(1.242,1.519) |
| Bladder (C67) | < 80/<75 | 476365 | 1336 | 3598482 | 0.37 | 0.592(0.553,0.634) | 0.9(0.84,0.964) | 0.917(0.855,0.982) |
|  | 80–85/75–80 | 401285 | 1622 | 3050723 | 0.53 | 0.847(0.794,0.904) | 0.904(0.847,0.964) | 0.91(0.853,0.971) |
|  | 85–90/80–85 | 436958 | 2085 | 3320756 | 0.63 | 1(Ref.) | 1(Ref.) | 1(Ref.) |
|  | 90–95/85–90 | 324680 | 1633 | 2457056 | 0.66 | 1.059(0.993,1.13) | 1.007(0.944,1.075) | 0.998(0.936,1.065) |
|  | 95–100/90–95 | 181355 | 841 | 1364177 | 0.62 | 0.984(0.908,1.066) | 0.994(0.917,1.077) | 0.977(0.902,1.059) |
|  | ≥ 100/ ≥ 95 | 134861 | 596 | 1003438 | 0.59 | 0.95(0.867,1.041) | 1.125(1.027,1.233) | 1.096(1,1.201) |
| Brain and central nervous system (C70-C72) | < 80/<75 | 476365 | 461 | 3601453 | 0.13 | 0.697(0.618,0.786) | 0.931(0.824,1.051) | 0.932(0.824,1.053) |
|  | 80–85/75–80 | 401285 | 496 | 3054597 | 0.16 | 0.884(0.785,0.995) | 0.943(0.837,1.061) | 0.943(0.837,1.061) |
|  | 85–90/80–85 | 436958 | 611 | 3326261 | 0.18 | 1(Ref.) | 1(Ref.) | 1(Ref.) |
|  | 90–95/85–90 | 324680 | 520 | 2461117 | 0.21 | 1.15(1.024,1.293) | 1.112(0.989,1.25) | 1.111(0.989,1.249) |
|  | 95–100/90–95 | 181355 | 281 | 1366133 | 0.21 | 1.12(0.973,1.29) | 1.085(0.942,1.25) | 1.083(0.94,1.248) |
|  | ≥ 100/ ≥ 95 | 134861 | 170 | 1004954 | 0.17 | 0.922(0.778,1.093) | 0.958(0.808,1.136) | 0.953(0.803,1.131) |
| Thyroid (C73) | < 80/<75 | 476365 | 2966 | 3590871 | 0.83 | 1.146(1.086,1.21) | 0.746(0.705,0.79) | 0.756(0.714,0.801) |
|  | 80–85/75–80 | 401285 | 2234 | 3046683 | 0.73 | 1.018(0.961,1.078) | 0.941(0.888,0.997) | 0.948(0.895,1.004) |
|  | 85–90/80–85 | 436958 | 2390 | 3317734 | 0.72 | 1(Ref.) | 1(Ref.) | 1(Ref.) |
|  | 90–95/85–90 | 324680 | 1778 | 2454832 | 0.72 | 1.005(0.946,1.069) | 1.048(0.985,1.114) | 1.042(0.98,1.108) |
|  | 95–100/90–95 | 181355 | 1086 | 1362352 | 0.80 | 1.107(1.03,1.189) | 1.122(1.045,1.206) | 1.111(1.034,1.194) |
|  | ≥ 100/ ≥ 95 | 134861 | 875 | 1001780 | 0.87 | 1.213(1.122,1.31) | 1.129(1.044,1.22) | 1.109(1.026,1.199) |
| Hodgkin's lymphoma (C81) | < 80/<75 | 476365 | 30 | 3602254 | 0.01 | 0.523(0.334,0.819) | 0.699(0.445,1.097) | 0.665(0.421,1.048) |
|  | 80–85/75–80 | 401285 | 39 | 3055482 | 0.01 | 0.802(0.53,1.212) | 0.847(0.56,1.28) | 0.831(0.549,1.257) |
|  | 85–90/80–85 | 436958 | 53 | 3327185 | 0.02 | 1(Ref.) | 1(Ref.) | 1(Ref.) |
|  | 90–95/85–90 | 324680 | 34 | 2462034 | 0.01 | 0.867(0.563,1.333) | 0.84(0.546,1.292) | 0.85(0.553,1.309) |
|  | 95–100/90–95 | 181355 | 18 | 1366585 | 0.01 | 0.826(0.484,1.411) | 0.816(0.478,1.393) | 0.833(0.487,1.425) |
|  | ≥ 100/ ≥ 95 | 134861 | 19 | 1005165 | 0.02 | 1.185(0.702,2.002) | 1.267(0.749,2.141) | 1.301(0.767,2.208) |
| Non- Hodgkin's lymphoma (C82-C86, C96) | < 80/<75 | 476365 | 692 | 3600673 | 0.19 | 0.628(0.57,0.692) | 0.848(0.77,0.935) | 0.829(0.752,0.915) |
|  | 80–85/75–80 | 401285 | 870 | 3053392 | 0.28 | 0.931(0.85,1.019) | 0.991(0.905,1.085) | 0.983(0.898,1.076) |
|  | 85–90/80–85 | 436958 | 1018 | 3324820 | 0.31 | 1(Ref.) | 1(Ref.) | 1(Ref.) |
|  | 90–95/85–90 | 324680 | 851 | 2460173 | 0.35 | 1.13(1.032,1.238) | 1.092(0.997,1.196) | 1.099(1.003,1.204) |
|  | 95–100/90–95 | 181355 | 473 | 1365633 | 0.35 | 1.132(1.015,1.263) | 1.104(0.99,1.232) | 1.119(1.003,1.248) |
|  | ≥ 100/ ≥ 95 | 134861 | 320 | 1004559 | 0.32 | 1.043(0.919,1.182) | 1.099(0.969,1.246) | 1.119(0.986,1.27) |
| Multiple myeloma (C90) | < 80/<75 | 476365 | 561 | 3600916 | 0.16 | 0.677(0.607,0.755) | 0.946(0.848,1.056) | 0.948(0.849,1.059) |
|  | 80–85/75–80 | 401285 | 613 | 3054071 | 0.20 | 0.872(0.784,0.969) | 0.933(0.839,1.038) | 0.934(0.839,1.039) |
|  | 85–90/80–85 | 436958 | 766 | 3325338 | 0.23 | 1(Ref.) | 1(Ref.) | 1(Ref.) |
|  | 90–95/85–90 | 324680 | 588 | 2460801 | 0.24 | 1.038(0.932,1.155) | 0.997(0.896,1.11) | 0.997(0.896,1.111) |
|  | 95–100/90–95 | 181355 | 355 | 1365846 | 0.26 | 1.129(0.996,1.281) | 1.102(0.971,1.25) | 1.102(0.972,1.251) |
|  | ≥ 100/ ≥ 95 | 134861 | 230 | 1004700 | 0.23 | 0.996(0.86,1.154) | 1.065(0.919,1.235) | 1.064(0.918,1.234) |
| Leukemia (C91-C95) | < 80/<75 | 476365 | 479 | 3601406 | 0.13 | 0.706(0.627,0.796) | 0.951(0.844,1.072) | 0.936(0.829,1.056) |
|  | 80–85/75–80 | 401285 | 541 | 3054547 | 0.18 | 0.94(0.838,1.054) | 0.997(0.889,1.119) | 0.993(0.885,1.114) |
|  | 85–90/80–85 | 436958 | 627 | 3326107 | 0.19 | 1(Ref.) | 1(Ref.) | 1(Ref.) |
|  | 90–95/85–90 | 324680 | 515 | 2461159 | 0.21 | 1.11(0.988,1.247) | 1.074(0.956,1.207) | 1.078(0.959,1.211) |
|  | 95–100/90–95 | 181355 | 298 | 1366135 | 0.22 | 1.158(1.009,1.329) | 1.136(0.989,1.304) | 1.145(0.997,1.315) |
|  | ≥ 100/ ≥ 95 | 134861 | 215 | 1004831 | 0.21 | 1.137(0.974,1.327) | 1.207(1.033,1.409) | 1.219(1.043,1.425) |
| Prostate (C61) | < 80/<75 | 173305 | 3065 | 1268720 | 2.42 | 0.739(0.707,0.772) | 0.814(0.779,0.851) | 0.842(0.805,0.88) |
|  | 80–85/75–80 | 205046 | 4442 | 1531635 | 2.90 | 0.884(0.85,0.92) | 0.922(0.886,0.959) | 0.932(0.896,0.97) |
|  | 85–90/80–85 | 224247 | 5509 | 1679807 | 3.28 | 1(Ref.) | 1(Ref.) | 1(Ref.) |
|  | 90–95/85–90 | 170141 | 4271 | 1269007 | 3.37 | 1.027(0.987,1.069) | 0.995(0.956,1.036) | 0.987(0.949,1.028) |
|  | 95–100/90–95 | 86539 | 2210 | 641665 | 3.44 | 1.054(1.003,1.107) | 1.041(0.991,1.093) | 1.026(0.976,1.078) |
|  | ≥ 100/ ≥ 95 | 59415 | 1294 | 437324 | 2.96 | 0.908(0.855,0.965) | 1.038(0.977,1.102) | 1.018(0.958,1.082) |
| Testis (C62) | < 80/<75 | 173305 | 32 | 1277416 | 0.03 | 0.882(0.564,1.38) | 0.946(0.605,1.481) | 0.955(0.608,1.501) |
|  | 80–85/75–80 | 205046 | 49 | 1545057 | 0.03 | 1.121(0.753,1.668) | 1.148(0.771,1.71) | 1.15(0.772,1.714) |
|  | 85–90/80–85 | 224247 | 48 | 1696559 | 0.03 | 1(Ref.) | 1(Ref.) | 1(Ref.) |
|  | 90–95/85–90 | 170141 | 49 | 1281893 | 0.04 | 1.35(0.906,2.009) | 1.331(0.894,1.982) | 1.331(0.894,1.983) |
|  | 95–100/90–95 | 86539 | 16 | 648246 | 0.02 | 0.87(0.494,1.532) | 0.87(0.494,1.532) | 0.87(0.493,1.534) |
|  | ≥ 100/ ≥ 95 | 59415 | 12 | 441179 | 0.03 | 0.956(0.508,1.8) | 1.04(0.552,1.958) | 1.039(0.55,1.962) |
| Breast (C50) | < 80/<75 | 303060 | 3096 | 2313451 | 1.34 | 1.009(0.955,1.066) | 0.834(0.787,0.884) | 0.843(0.795,0.894) |
|  | 80–85/75–80 | 196239 | 2019 | 1502723 | 1.34 | 1.013(0.953,1.076) | 0.964(0.907,1.025) | 0.965(0.908,1.026) |
|  | 85–90/80–85 | 212711 | 2153 | 1622728 | 1.33 | 1(Ref.) | 1(Ref.) | 1(Ref.) |
|  | 90–95/85–90 | 154539 | 1604 | 1174177 | 1.37 | 1.03(0.966,1.099) | 1.054(0.988,1.124) | 1.055(0.989,1.125) |
|  | 95–100/90–95 | 94816 | 1013 | 714551 | 1.42 | 1.07(0.993,1.153) | 1.105(1.025,1.191) | 1.107(1.027,1.193) |
|  | ≥ 100/ ≥ 95 | 75446 | 836 | 561037 | 1.49 | 1.126(1.039,1.219) | 1.137(1.049,1.231) | 1.139(1.051,1.235) |
| Cervix (C53) | < 80/<75 | 303060 | 584 | 2322770 | 0.25 | 0.984(0.868,1.116) | 1.019(0.894,1.161) | 1.019(0.893,1.163) |
|  | 80–85/75–80 | 196239 | 375 | 1508998 | 0.25 | 0.973(0.846,1.119) | 0.982(0.854,1.129) | 0.981(0.853,1.129) |
|  | 85–90/80–85 | 212711 | 416 | 1629205 | 0.26 | 1(Ref.) | 1(Ref.) | 1(Ref.) |
|  | 90–95/85–90 | 154539 | 341 | 1178971 | 0.29 | 1.133(0.982,1.307) | 1.128(0.977,1.302) | 1.127(0.977,1.301) |
|  | 95–100/90–95 | 94816 | 208 | 717632 | 0.29 | 1.135(0.961,1.34) | 1.128(0.955,1.332) | 1.126(0.953,1.331) |
|  | ≥ 100/ ≥ 95 | 75446 | 186 | 563437 | 0.33 | 1.292(1.087,1.535) | 1.289(1.084,1.532) | 1.279(1.075,1.522) |
| Uterus (C54) | < 80/<75 | 303060 | 400 | 2323496 | 0.17 | 0.845(0.73,0.977) | 0.627(0.537,0.731) | 0.651(0.557,0.76) |
|  | 80–85/75–80 | 196239 | 287 | 1509327 | 0.19 | 0.933(0.797,1.093) | 0.864(0.737,1.012) | 0.875(0.746,1.025) |
|  | 85–90/80–85 | 212711 | 332 | 1629396 | 0.20 | 1(Ref.) | 1(Ref.) | 1(Ref.) |
|  | 90–95/85–90 | 154539 | 282 | 1179034 | 0.24 | 1.174(1.002,1.376) | 1.217(1.038,1.426) | 1.208(1.031,1.417) |
|  | 95–100/90–95 | 94816 | 162 | 717752 | 0.23 | 1.108(0.918,1.337) | 1.166(0.966,1.407) | 1.148(0.95,1.386) |
|  | ≥ 100/ ≥ 95 | 75446 | 218 | 563217 | 0.39 | 1.902(1.603,2.256) | 1.926(1.624,2.285) | 1.875(1.577,2.228) |
| Ovary (C56) | < 80/<75 | 303060 | 901 | 2322456 | 0.39 | 0.857(0.778,0.945) | 0.875(0.79,0.968) | 0.886(0.8,0.982) |
|  | 80–85/75–80 | 196239 | 630 | 1508723 | 0.42 | 0.92(0.827,1.023) | 0.925(0.832,1.029) | 0.93(0.836,1.035) |
|  | 85–90/80–85 | 212711 | 739 | 1628765 | 0.45 | 1(Ref.) | 1(Ref.) | 1(Ref.) |
|  | 90–95/85–90 | 154539 | 550 | 1178724 | 0.47 | 1.029(0.922,1.149) | 1.027(0.92,1.147) | 1.023(0.916,1.142) |
|  | 95–100/90–95 | 94816 | 386 | 717533 | 0.54 | 1.189(1.052,1.345) | 1.185(1.048,1.34) | 1.176(1.039,1.331) |
|  | ≥ 100/ ≥ 95 | 75446 | 334 | 563232 | 0.59 | 1.318(1.158,1.5) | 1.316(1.156,1.498) | 1.298(1.14,1.478) |

^a^ Model 1, Non-adjusted. ^b^ Model 2, adjusted for age and sex. ^c^ Model 3, Adjusted for age, sex, smoking, alcohol drinking, regular exercise, low income, previous history of hypertension and dyslipidemia. Abbreviations: DM, diabetes mellitus; BMI, body mass index; WC, waist circumference; HR, hazard ratio; CI, confidential interval; NGF, normal fasting glucose; IFG, impaired fasting glucose.
